# Identifying the unmet supportive care needs of individuals affected by testicular cancer: a systematic review

**DOI:** 10.1007/s11764-022-01219-7

**Published:** 2022-07-04

**Authors:** R. Doyle, P. Craft, M. Turner, C. Paterson

**Affiliations:** 1 Faculty of Health, School of Nursing, Midwifery & Public Health, University of Canberra, Bruce, ACT 2601 Australia; 2 Prehabilitation, Activity, Cancer, Exercise and Survivorship (PACES) Research Group, University of Canberra, Bruce, ACT Australia; 3 School of Medicine, Australian National University, Canberra, ACT Australia; 4 Robert Gordon University, Aberdeen, Scotland, UK; 5 ACT, Canberra Health Services, SYNERGY Nursing & Midwifery Research Centre, ACT Health Directorate, Canberra Hospital, Level 3, Building 6, GPO Box 825, Canberra, 2601 Australia

**Keywords:** Patient care, Supportive care needs, Testicular cancer, Testicular neoplasm, Integrative review, Systematic

## Abstract

**Purpose:**

To critically appraise studies to identify experiences of unmet supportive care needs of individuals affected by testicular cancer.

**Methods:**

A registered priori systematic review was conducted in accordance with Preferred Reporting Items for Systematic Reviews and Meta-Analyses (PRISMA) guidelines. CINAHL, PsycINFO, and MEDLINE were searched for quantitative, qualitative, and mixed methods studies using a wide range of search terms. All articles were double screened according to a pre-determined eligibility criterion. Reference lists of the final included studies were checked for further eligible studies. The review process was managed using Covidence systematic review software. Data from the studies were extracted, methodological quality appraisal conducted, and a narrative synthesis conducted.

**Results:**

Of the 72 papers identified, 36 studies were included. In descending order of frequency of need, psychological needs were identified in 26/36, physical needs 18/36, interpersonal/intimacy needs 19/36, health system/information needs 11/36, cognitive needs 9/36, social needs 7/36, and of equal frequencies counts of 4/36 for family, practical, and patient-clinician information needs. Only one study explored spiritual needs and no daily living needs were identified.

**Conclusions:**

The experience of needs varied in terms of frequency and distress which were commonly influenced by the age of the individual across the cancer care continuum persisting after 1-year post-treatment.

**Implications for Cancer Survivors:**

When caring for individuals affected by testicular cancer, clinicians are encouraged to take a holistic lens to cancer care, particularly to explore issue or concerns that young men affected by testicular cancer might be embarrassed or reticent to discuss.

**Supplementary Information:**

The online version contains supplementary material available at 10.1007/s11764-022-01219-7.

## Introduction

Testicular cancer is the 26th most commonly diagnosed cancer worldwide [1]. Evidence has underscored that testicular cancer is the most prevalent type of cancer diagnosed among young men aged 15–35 years [2] with 74,458 cases diagnosed globally in 2020 [3]. Testicular cancer is highly curable with survival rates estimated above 90% largely attributed to the introduction of platinum-based chemotherapies [4] resulting in an increased number of survivors. Treatments include orchiectomy, retroperitoneal lymph node dissection, chemotherapy, and radiotherapy, [5] all of which are associated to their own unique profile of concerns with implications for rehabilitation and supportive care [6].

Supportive care is defined as a holistic term used to describe a person-centred approach to the delivery of oncology services for individuals diagnosed with cancer to meet their informational, spiritual, psychological, social, or physical needs across the cancer care continuum [7]. Healthcare professionals and researchers have a growing awareness of the importance to identify gaps in supportive care experiences for people affected by cancer. Though the timely identification of unmet needs, planning and delivery of cancer services can be targeted to improve patients’ overall health-related quality of life and recovery [7]. To date, a growing number of systematic reviews have examined the unmet needs in various cancer populations such as prostate [8], bladder [9], gynaecological [10], kidney [11], penile [12], breast [13], and colorectal [14], including older populations affected by cancer [13]. However, none of these existing evidence synthesis studies provides any clinical insight into the unique needs of young men affected by testicular cancer [6, 15, 16]. The life expectancy among men diagnosed with testicular cancer is about 30–50 years after treatment. Due to high survival rates, minimising the adverse effects of treatment is a major issue and of central importance. Unmet supportive care needs are associated with quality-of-life outcomes in people with cancer, and therefore, supportive care is considered a modifiable factor for research and service provision [17].

Evidence has demonstrated that men affected by testicular cancer commonly report at least one unmet supportive care need despite routine clinical follow-up [15, 16, 18]. Moreover, unmet supportive care needs have been reported to persist up to 1 year after treatment and correlate with anxiety and depression [19]. These young men may have enduring physical and psychological needs related to diagnosis and treatments comparative to their youth. Existing studies have reported enduring and long-lasting effects from treatment which include problems related to infertility, altered neurological and respiratory function, problems in securing life insurance and employment, psychological distress (such as fear of cancer recurrence), altered masculinity/body image, concerns related to chemotherapy-induced alopecia, and challenges with intimacy and relationships [6, 7, 15, 16, 19]. Physical needs are prevalent in testicular cancer survivors, who on average may experience 4.5 physical symptoms (SD = 4.4; range, 1–28) [20]. Existing studies have identified that the physical needs among testicular cancer survivors are associated with unemployment, age, low socioeconomic status, and anxiety and depression [20, 21]. Furthermore, the psychological/emotional needs of individuals affected by testicular cancer were also found to be high with on average 1.4 psychological unmet needs [20]. Emotional needs are related to emotional functioning, depression [22], hopeless coping style [23], and cancer-related masculinity threat [24]. Several studies [6, 8, 16, 18, 19] have been conducted to explore the unmet supportive care needs of men affected by testicular cancer. To date, there has not been a systematic review to critically appraise the existing evidence to identify the classification of supportive care needs among men affected by testicular cancer to inform the planning and development of cancer services.

## Research questions

This systematic review set out to address the following research questions:

1. What are the unmet supportive care needs of individuals affected by testicular cancer?
2. What are the most frequently reported individual domains of unmet need in individuals affected by testicular cancer?

## Methods

### Design

This integrative systematic review was conducted and reported according to the Preferred Reporting Items for Systematic Reviews and Meta-Analysis (PRISMA) guidelines [25, 26] (see supplementary Table 1). This review also followed a registered priori systematic review protocol available from: PROSPERO: https://www.crd.york.ac.uk/prospero/display_record.php?RecordID=292072.

### Eligibility criteria

#### Types of studies

Inclusion:

- All qualitative, quantitative, and mixed methods studies irrespective of research design.
- All studies published in the English language within the last 10 years.

Exclusion:

- Commentaries, editorials, and studies where unmet supportive care needs are not reported were excluded.

#### Types of participants

Inclusion:

- Participants diagnosed with testicular cancer, irrespective of cancer stage or treatment.
- Studies conducted with patients in mixed cancer groups, except where separate subgroup analyses of only testicular cancer participants were reported.

#### Types of outcomes

Inclusion:

The primary outcome of this review was non-oncological outcomes related to unmet supportive care needs. The Supportive Care Needs Framework [27] guided the classification of supportive care needs. Outcomes specifically are related to the measurement of unmet supportive care needs (e.g. the Supportive Care Needs Survey [28]) and qualitative experiences, informed by the definition of supportive care (see Table 1 for classification).

**Table 1:**
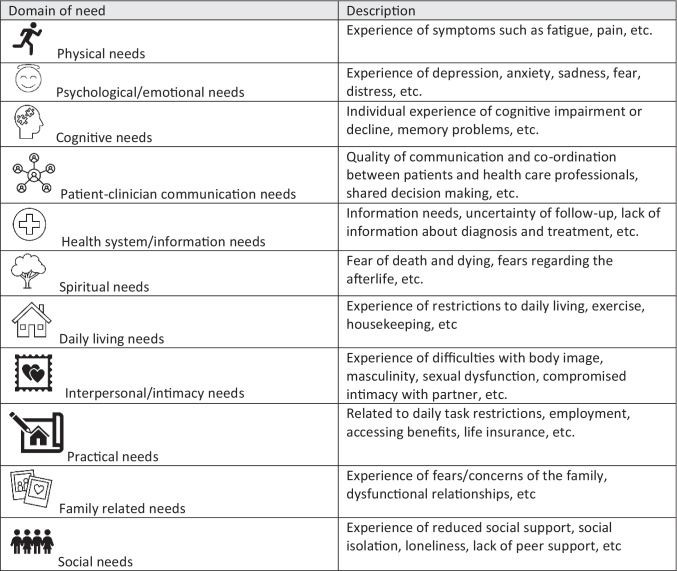
Classification of supportive care needs

### Literature search

The APA PsycINFO, CINAHL, and MEDLINE databases were searched in November 2021 for relevant studies published from 2002 onwards. To capture as many studies as possible, the database search architecture utilised a wide range of keywords and subject headings. Limiters were placed on all searches for peer-review and English language. A full record of the database searches is included in Supplementary Table 2. The reference lists of all the articles included were searched to locate additional relevant studies. Citations were managed with Endnote 20 and imported into Covidence systematic review software to facilitate the study selection process.

### Selection of studies

Following de-duplication, titles and abstracts were double screened independently by reviewers for eligibility, and any disagreements were resolved by discussion. Full texts were then retrieved, double screened by reviewers, and linked multiple records of the same study together. Any disagreements were resolved by discussion. The process of the selection of studies was conducted in Covidence systematic review software.

### Data extraction and management

Data extraction was performed on the retained full-text studies meeting the inclusion criteria. The data was extracted by one reviewer and independently quality checked by a second reviewer. The data extraction tables were developed and tested on a small sample of studies and then further refined through discussion among the reviewers. The first table of data extraction included information on the purpose, setting, country, sample size, participant characteristics, sampling used, response rate, attrition, design, time points, and data collection tools. The second data extraction table related to the supportive care needs outcome data according to the classification of supportive care needs (see Table 1).

### Assessment of risk of bias in included studies

The final retrained full-text studies all underwent a methodological quality assessment. None of the studies was excluded based upon their methodological quality score to enable a comprehensive overview of the current state of the evidence. The methodological quality assessment was conducted using the Mixed Methods Assessment Tool (MMAT) [29]. The MMAT tool was selected because it enables a plethora of study designs to methodological appraised given the integrative review design. This assessment tool enables critical appraisal of all qualitative, quantitative, and mixed methods studies. Each domain of assessment is rated against, “no”, “yes”, and “unclear”. Methodological quality assessment was performed by one reviewer and quality checked by a second reviewer.

### Data synthesis

This integrative review used a narrative synthesis [30]. The steps in the narrative synthesis involved (1) data reduction by tabulation, (2) data comparison between studies, and finally, (3) drawing conclusions. This process involved reading the full papers multiple times, linking together similarities and differences between the studies, and quality checking with the primary sources. The data reduction involved delineation of the classification by domain of unmet need within the tabulated data. The data comparison phase involved the reviewers’ identifying patterns and themes through counting and clustering and making comparisons and contrasting the study findings. Finally, the drawing of conclusions and verification involved a subgroup analysis to inform a comprehensive understanding of the topic, which was verified with the primary sources data for accuracy throughout the process. The data synthesis was conducted by two reviewers and consulted with a third reviewer. The reviewers were multidisciplinary healthcare professionals in cancer care.

## Results

**Fig. 1:**
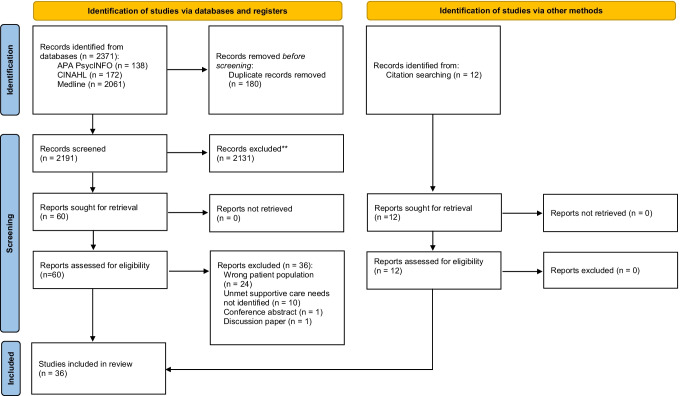
PRISMA 2020 flow diagram for new systematic reviews which included searches of databases, registers, and other sources

The initial search yields 2383 results (see Fig. 1). A total of 72 full-text articles were assessed, and 36 articles were excluded with reasons (see Fig. 1). A total of 36 studies fully met the inclusion criteria of which there were five qualitative [21, 31–34], 30 quantitative [15, 18, 20, 22–24, 35–58], and one mixed methods [59] which underscores that this is a developing evidence base (see Table 2). Studies were conducted in the UK (5), the USA (5), Canada (4), Germany (4), Norway (4), Australia (3), Denmark (2), Italy (2), the Netherlands (2), Turkey (2), Greece (1), Lebanon (1), Serbia (1), and Sweden (1). The sample sizes of the included studies varied widely; 16 studies had < 100 participants, 17 studies had ≥ 100 participants, two studies had > 500 participants, and one failed to report how many participants were included [33]. The average age of study participants varied from 25.1 to 44.4 years, and most of the participants had localised disease compared to metastatic disease. Treatments also varied, but most participants were treated by either orchiectomy or orchiectomy and chemotherapy. Although some underwent surveillance, radiotherapy, and/or retroperitoneal lymph node dissection (RPLND) were reported, most of the participants were married, were in full-time employment, and had at least secondary education or higher. Therefore, the participants in this review are not representative of other minority groups (see Table 3 for the results of the methodological quality assessment). Most of the studies were cross-sectional in design and therefore provide little information about how supportive care needs change over time. The studies had small sample sizes and used convenience sampling approaches.

**Table 2:** Overview of the included studies

Author and Year Country	Purpose	Setting	Sample size	Participants	Sampling	Response rate	Attrition	Design	Time points	Data collection tools
Alacaciouglu et al., [22]. Turkey	To determine the prevalence of anxiety depression and sexual satisfaction in TCS and compare rates with healthy controls.	Izmir Katip Celebi University Atatürk Research and Training Hospital Clinic, Medical Oncology.	N=41 N=38 healthy controls.	**Clinical:** 39% had a family history of cancer. No further details reported. **Demographics:** Mean age (range): 38 (26-42). post-graduate education: 24.4%.	Convenience	Not reported.	N/A.	Cross sectional survey.	1.	**Questionnaires:** Demographics: Unspecified questionnaire. Depression and anxiety: Hospital Anxiety and Depression Scale (HADS). Sexual functioning: Golombuk-Rust of Sexual Satisfaction (GRISS). Quality of life: European Organisation for Research of Treatment of Cancer Questionnaires Quality of Life – C30 (EORTC-QoL-C30).
Amidi et al., [35]. Denmark	To investigate the experiences of cognitive impairment post-treatment in TCS.	Department of Oncology, Aarhus University Hospital.	N=66. N=25 healthy controls.	**Clinical:** Unilateral orchiectomy – 100% (N=66). I – 70% (N=46). II – 26% (N=17). III – 4% (N=3). **Demographics:** Mean age (SD) - 36.8 years (10.9). Mean number of years of education (SD) – 14.2 (2.3).	Convenience	70%.	N/A.	Cross sectional survey.	1.	**Questionnaires and clinical data:** Reaction time: MOART Reaction and Movement Time Panel. Processing speed: Wechsler Adult Intelligence Scale version 4 (Ws6AIS-IV)—Coding Trail-Making Test Part A (TMT-A). Attention and working memory: WAIS-IV—Digit span and the Paced Auditory Serial Addition Test (PASAT). Verbal learning and memory: Rey Auditory Verbal Learning Test (RAVLT). Verbal fluency: Controlled Oral Word Association (COWAT). Executive functioning: Trail-Making Test Part B (TMT-B) Wisconsin Card Sorting Test (WCST). Premorbid intelligence functioning: WAIS Vocabulary subtest. Psychological distress: The Hospital Anxiety and Depression Scale (HADS) Perceived Stress Scale (PSS). Post-traumatic stress symptoms: Impact of Events Scale—Revised (IES-R). Cognitive complaints: Cognitive Failures Questionnaire (CFQ). Medical variables: Extracted from medical records. **Biological data:** Serum cortisol, Plasma interleukin-6, Tumour Necrosis Factor alpha, C. reactive protein.
Amidi et al., [36] Denmark.	To determine the frequency of cognitive impairment in TCS.	1 – The Department of Oncology, Aarhus University Hospital.	N=72.	**Clinical:** Chemotherapy - (3/4 cycles, BEP) 50% (N=36). No chemotherapy – 50% (N=36). Radiotherapy – 18.1% (N=13). No radiotherapy – 81.9% (N=59). I –43.1% (N=31). II – 44.4% (N=32). III – 8.3% (N=6). IV – 4.2% (N=3). **Demographics:** Mean age (SD [range]) – 40.1 (9.7) [24 -70]. Mean years of education (SD) [range] – 13.3 (3.0) [7-18].	Convenience	31.2%.	N/A.	Cross sectional survey.	1.	**Questionnaires:** Attention and working memory: Wechsler Adult Intelligence Scale III Digit Span. Wechsler Adult Intelligence Scale III Letter-Number Sequencing. Wechsler Adult Intelligence Scale III Arithmetic. Processing speed: Wechsler Adult Intelligence Scale III Coding. Wechsler Adult Intelligence Scale III Symbol Search. Trail Making Test – Part A. Verbal fluency: Primary Mental Abilities Test. Verbal learning and memory: Rey Auditory Verbal Learning Test. Visual learning and memory: Rey Complex Figure and Recognition Trial (1^st^ trial). Rey Complex Figure and Recognition Trial (total). Executive functioning: Trail Making Test – Part B.
Batehup et al., [37] UK	To investigate the unmet supportive care needs of survivors of breast, colorectal and testicular cancers and determine their frequency.	Not reported.	N=75 at T0. N=41 at T2.	**Clinical:** Surgery only – 14.9% (N=13). Surgery and chemotherapy – 80.5% (N=70). Surgery and radiotherapy - 3.4% (N=3). Chemotherapy only – 1.1% (N=1). **Demographics:** Mean age (SD) – 39.1 (12.2).	Convenience	72%.	44.8% at T2 (8 months).	Prospective longitudinal survey.	4	**Questionnaire:** Unmet supportive care needs: Modified 25 question Cancer Survivors Unmet Needs Survey (CaSUN).
Bender et al., [15] Canada	To investigate the unmet supportive care needs of TCS, assess TCS preferences for receiving online support	1 - Princess Margaret Hospital, Toronto.	N=204.	**Clinical:** Surveillance – 55.9% (N=114) Radiation – 8.3% (N=17) Chemotherapy – 17.6% (N=36) RPLND – 2.5% (N=5) Chemotherapy and RPLND - 14.7% (N=30) Radiation and chemotherapy – 0.5% (N=1) Radiation, chemotherapy and RPLND – 0.5% (N=1) **Demographics:** Mean age (SD) – 35.6 (10.5).	Convenience	71.3%.	N/A.	Cross sectional survey.	1	**Questionnaire:** Sociodemographic characteristics: Age, Education, Income, Employment status, Place of birth, First language. Unmet supportive care needs: Altered version of the Cancer Survivors Unmet Needs (CaSUN) instrument. Use of computers, the internet and social media: “Seven categorical and three-point response (yes, no, unsure) questions developed based on previous research by investigators JLB and DW.”
Brand et al., [38] UK	To evaluate the effect of active surveillance of testicular cancer on the sexual functioning of people	Not specified.	N=21.	**Clinical:** Stage one germ cell testicular cancer with or without lymphovascular invasion -21 people (100%). Orchiectomy – 100% (N=21). **Demographics:** Not reported.	Convenience	Not specified.	N/A.	Prospective longitudinal study.	2	**Two-part questionnaire:** Part 1: Sexual function: “Introductory questions”. Brief Male Sexual Function Inventory for Urology. Part 2: Information seeking:
Bumbasirevic et al., [39] Serbia	To investigate sexual functioning, physical symptoms, HRQoL, and depression among TCS	1 - Clinic of Urology, Clinical Center of Serbia.	N=202.	**Clinical:** Radical orchiectomy and adjuvant chemotherapy - 91.5% (N=185). Radical orchiectomy adjuvant chemotherapy retroperitoneal lymph node dissection - 8.5% (N=17). **Demographics:** Age not reported.	Convenience	96%.	N/A.	Cross sectional study	1.	**Questionnaires:** Health related quality of life: Short Form 36 (SF-36) European Organization for Research Treatment of Cancer Quality of Life Questionnaire (EORTC QLQ-C30). Feeling and attitudes to general depressive status: The Beck Depression Inventory (BDI). Sexual functioning: Generic questionnaire comprising of nine yes/no answer questions about erectile and ejaculatory function, sexual drive, assessment of sexual life before and after treatment.
Carpentier et al., [31] USA	To explore the romantic and sexual relationships of young people after their experience with testicular cancer.	1 - Indiana University Simon Cancer Center.	N=21.	**Clinical:** Treatment not reported. **Demographics:** Age (mean): 27.1 years.	Purposive	Not specified.	N/A.	Phenomenological study.	1.	**Semi structured interview:** Based on Ferrell et al’s model of quality of life in cancer survivorship. Questions focused on: social and relational quality of life.
Darabos and Hoyt, [40] USA	To investigate future worry in the context of perceived stress	Not specified.	N=171.	**Clinical:** Radical inguinal orchiectomy – 73.1% (N=125). Bilateral orchiectomy – 7% (N=12). Retroperitoneal lymph node dissection – 24% (N=41). Chemotherapy – 53.2% (N=91). Radiation – 15.2% (N=26). Other – 8.2% (N=14). **Demographics:** Mean age (SD)- 25.21 (3.33).	Convenience	59%.	Not specified.	Cross sectional study.	1.	**Questionnaire:** Cancer related worry: Two-item future perspective subscale of the European Organisation for Research and Treatment of Cancer Quality of Life Questionnaire for Testicular Cancer (EORTC QLQ-TC26). Perceived stress: 10-item Perceived Stress Scale. Physical wellbeing: Seven-item physical well-being Subscale of the Functional Assessment of Cancer Therapy General (FACT-G).
De Padova et al., [41] Italy	To explore the perceptions of TCS and their carers regarding survivorship and compare them to the perceptions of healthcare providers.	1 - Istituto Tumori Romagna – IRST.	Patients – N=29. Carers – N=14. Healthcare providers – N=42.	**Clinical:** Surveillance – 21% (N=6). CT +/- RT +/- RPLND – 66% (N=19). RT only – 7% (N=2). RPLND only – 3% (N=1). Not answered – 3% (N=1). **Demographics:** Median age (range) - 36 (22- 60).	Convenience	Patients – 91% (20/32). Carers – 100% (14/14). Healthcare providers – 70% (42/60).	N/A.	Cross sectional survey.	1.	**Questionnaire:** Author developed questionnaire investigating: demographics, quality of life, role of healthcare providers/ services.
Dimitropoulos et al., 2016 Greece	To evaluate the sexual functioning of patients who were treated with retroperitoneal lymph node dissection post chemotherapy before and after treatment.	1 - Department of Urology, University Hospital od Larissa, Greece.	N=63.	**Clinical:** Time interval from last chemotherapy to PC-RPLND (weeks) - 6.2 (3.86). IIB – 7.5% (N=4). IIC – 28.3% (N=15). IIIA – 3.8% (N=2). IIIB – 50.1% (N=27). IIIC – 9.4% (N=5). **Demographic:** Mean age at diagnosis (SD): 30.4 (7.89).	Convenience	N/A.	Not reported.	Prospective longitudinal.	2.	**Questionnaires:** Sexual function: International Index for Erectile Function (IIEF). Demographics, medical History, sexual functioning
Kerns et al., [43] USA, UK and Canada	To analyse the relationship between cisplatin related adverse events and self-rated health, unemployment and disability in TCS	8 - University of Rochester, University of Pennsylvania, Indiana University, Dana Farber Cancer Institute, Memorial Sloan Kettering Cancer Center, M.D. Anderson), Princess Margaret Hospital and British Columbia Cancer Agency	N=1815.	**Clinical:** Seminoma – 24.6% (N=447). Non-seminoma or mixed germ cell tumour – 73.2% (N=1328). Germ cell tumour not otherwise specified – 2.2% (N=40). RPLND: Yes - 46.5% (N=836). No – 53.5% (N=962). **Demographics:** Median age: 37 (18 – 75)	Convenience	Not reported.	N/A.	Cross sectional.	1.	**Questionnaires:** Neuropathy: European Organization for Research and Treatment of Cancer Chemotherapy-Induced Peripheral Neuropathy (EORTC CIPN-20). Neurotoxicity: Scale for Chemotherapy-Induced Neurotoxicity (SCIN). Adverse events: Terminology Criteria for Adverse Events. Physical activity: Minnesota LTA questionnaire.
Martin et al., [21] UK	To describe the needs of TCS	Southampton General Hospital.	N=24	**Clinical:** Not reported. **Demographic:** Not reported.	Convenience	Not reported.	N/A.	Qualitative	4	Needs assessment participants were asked for “reasons why survivorship care services need to be improved, as well as causes of that antecedent” and a logic map created.
Matheson et al., [32] UK	To explore young men’s adjustment to survivorship of testicular cancer.	Three NHS hospitals in the south of England.	N=18.	**Clinical:** Surgery alone – 33% (N=6). Surgery + chemotherapy/or radiotherapy – 67% (N=12). **Demographics:** 20 – 24 years – 11% (N=2). 25 – 29 years – 17% (N=3). 30 – 34 years – 22% (N=4). 35 – 39 years – 22% (N=4). 40 – 45 years – 28% (N=5).	Convenience	33%.	22.2222%.	Longitudinal qualitative.	2	Semi-structured interviews
Nord et al., [44] Sweden	To determine if TCS experience more work loss	Not reported.	2146 people with testicular cancer. 8448 people in comparison group.	**Clinical:** Surveillance – 29% (N=605). Radiotherapy – 14% (N=300). Chemotherapy [1 course] – 29% (N=621). Chemotherapy [2 – 3 courses] – 6% (N=118). **Demographics:** Median age: 32.	Convenience	Not reported.	N/A.	Cohort	1.	Data from SWENOTECA (Swedish Norwegian Testicular Cancer Group) database, Causes of Death Register, Statistics Sweden and Social Insurance Agency database.
O’Carrigan et al., [45] Australia.	To examine the frequency of abnormal serum hormone levels in TCS and determine if the presence if hormone levels is associated with quality of life or psychological health.	Multiple centres (details unspecified).	N=100.	**Clinical:** Surgery – 94% (N=51). Chemotherapy – 72% (N=39). Radiotherapy – 15% (N=8). **Demographics:** Median age (years) (range) – 35 (19-64)	Convenience	N/A.	N/A.	Cross sectional survey.	1.	**Biological data:** Serum testosterone, serum follicle stimulating hormone, luteinizing hormone. **Questionnaires:** Depression and anxiety: Hospital anxiety and depression scale (HADS). Fatigue: Functional Assessment of Chronic Illness Therapy – Fatigue (FACTIF-F)
Oechsle et al., [20] Germany.	To investigate what symptoms that long term TCS commonly experience and find distressing	University Cancer Center Hamburg.	N=164.	**Clinical:** 98% (N=160). Orchiectomy only – 56% (N=92). Chemotherapy at first diagnosis – 70% (N=117). Radiotherapy at any time – 23% (N=37). **Demographic:** Not reported.	Convenience	61.1%.	N/A.	Cross sectional survey.	1.	**Questionnaire:** Symptom burden: Memorial Symptom Assessment Scale (MSAS-SF).
Pallotti et al., [46] Italy.	To investigate the sexual functioning of long term TCS who underwent chemotherapy and orchiectomy	Laboratory of Seminology—Sperm Bank “Loredana Gandini”.	N=241. Control N= 223.	**Clinical:** 100% chemotherapy and orchiectomy **Demographics:** Mean age at diagnosis (SD), (median) (range) - 31.3 (6.9) (31.0) (26 – 36).	Convenience	Not reported.	Not clearly reported.	Prospective longitudinal survey.	7.	**Questionnaire:** Erectile function: Index of Erectile Function 15 questionnaire (IIEF-15). **Biological data:** Subgroup of TCS underwent hormone testing of follicle stimulating hormone, luteinising hormone, testosterone.
Pühse et al., [47] Germany.	To investigate the effect of chronic pain on sexuality in TCS.	1 – University Hospital of Münster.	N=248.	**Clinical:** Chemotherapy – 45.8% (N=109). Radiation therapy – 17.2% (N=41). RPLND – 26.9% (N=64). Stage I – N=125, Stage II – N=71, Stage III – N=39 **Demographics:** Mean age at orchiectomy (SD) (range) – 35.2 (9.3) (19 – 69)	Convenience	58.2%.	N/A	Cross sectional survey.	1.	**Questionnaires:** Chronic pain: occurrence of phantom testis pain, phantom testis sensations, hallucinations. Erectile function: Abbreviated International Index of Erectile Function (IIEF-5).
Saab et al., [33] Lebanon.	To explore the experiences of Lebanese TCS.	1 - Unspecified clinic.	Not reported.	**Clinical:** Orchiectomy and chemotherapy – N=5. Orchiectomy alone – N=2. Orchiectomy and radiotherapy – N=1. **Demographic**: Mean age (range): (41 (32 – 50).	Purposive	Not reported.	N/A.	Phenomenological study.	1.	Semi-structured interview.
Shen et al., [59] Canada.	To explore the experiences of TCS	1 - Ambulatory urologic oncology clinics at Princess Margaret Cancer Centre.	Questionnaire N=90. Focus group N=7. Phone interview N=6.	**Clinical:** Surgery only – 70.8% (N=63). Surgery/chemotherapy – 21% (N=19). Surgery/radiotherapy – 5.6% (N=5). Surgery/chemotherapy/radiotherapy – 2.2% (N=2). **Demographics:** Age (years): 18 – 50 – 90% (N=81). 51 – 60 – 8.89% (N=8). 60 + – 1.11% (N=1).	Convenience	Questionnaire 59%. Focus group 7.8%. Phone interview 6.7%.	N/A.	Mixed methods.	1.	**Questionnaires:** Demographics and treatment, Self-reported questionnaire. Survivorship knowledge: Eight questions adapted from the Breast Cancer Survivors Knowledge of Disease and Treatment Questionnaire on a five-point Likert scale. Feelings of preparedness for transition to follow-up care: 4 items from the perceived preparedness for re-entry scale. Health related distress: Modified version of the medical outcomes study-health distress scale (MOS-HDS). Continuity of care: Modified version of the Patient Continuity of Care Questionnaire (PCCQ). **Interviews:** Focus group interview, Telephone interview
Skaali et al., [48] Norway	To evaluate the cognitive functioning of TCS.	2 - Norwegian Radium Hospital and Ullevål Hospital.	N=122	**Clinical: S**urveillance/radiotherapy only **Demographic:** Age at baseline (years), median [range] – 32.5 [19-60].	Convenience	64%.	5%.	Prospective longitudinal	2.	**Neuropsychological tests:** Intellectual functioning: Norwegian version of the National Adult Reading Test (NART). Trauma: Impact of Event Scale (IES). Fatigue: The Fatigue Questionnaire. Neurotoxicity: Scale for Chemotherapy-Induced Neurotoxicity (SCIN). Neuroticism: Eysenck Personality Questionnaire. Alcohol Use: CAGE Questionnaire. Learning/memory: Hopkins Verbal Learning Test – Revised (HVLT-R). Paired associates learning test (PAL). Attention/concentration/working memory: Spatial Working Memory Test (SWM). Choice Reaction Time (CRT). Motor function: Grooved Pegboard (GP). Psychomotor speed: Trail Making Test-A (TMT-A). Colour-Word Interference Test 1 + 2 (CW-1+2). Executive Function: Colour-Word Interference Test 3 + 4 (CW 3 + 4). Trail Making Test – B (TMT-B). Word Fluency (FAS). Stockings of Cambridge (SOC). Intra-Extra Dimensional (IED) set shift.
Skaali et al., [49] Norway	To determine the presence and frequency of self-reported cognitive issues in TCS treated with and without chemotherapy.	Not specified.	N=129 (at baseline). N=122 (at follow up.)	**Clinical:** Metastatic disease (TC Stage II -IV) – 28% (N=34). **Demographics:** Age at baseline - 32.5.	Convenience	64%.	5%.	Prospective longitudinal survey.	2.	**Semi structured interview investigating:** Self-reported cognitive problems Participants categorised their responses in either “very good” or “good” or “not so good” or “poor” in response to questions about the quality of their concentration and memory function at baseline and follow up. Concentration and memory problems before and/or after a TC diagnosis were explored and participants categorised their responses on an 11-point Likert scale. **Questionnaires:** Psychological response to TC diagnosis The Impact of Event Scale (IES). Fatigue: The Fatigue Questionnaire. Neurotoxicity: Scale for Chemotherapy-Induced Neurotoxicity (SCIN). Neuroticism: Eysenck Personality Questionnaire (EPQ-18). Alcohol use: Four-item version of the CAGE questionnaire. Intellectual functioning: Norwegian version of the National Adult Reading Test (NART). Attention, concentration and working memory: Spatial Working Memory test and the Choice Reaction Time test. Learning and memory: Hopkins Verbal Learning Test–Revised Paired. Associates Learning test. Speed of information processing: Trail Making Test Part A. Color–Word Interference Test Parts 1 and 2. Executive functions: Color–Word Interference Test Parts 3 and 4. Trail Making Test B. Word Fluency (FAS) test. Stockings of Cambridge test. Intra-Extra Dimensional Set Shift test. Motor function: Grooved Pegboard test.
Skaali et al., [50] Norway	To determine the prevalence of cancer related distress in newly diagnosed TCP	2 - Norwegian Radium Hospital or Ullevaal University Hospital.	N=135	**Clinical**: Seminoma – 53% (N=71). Non-seminoma – 47% (N=64). Stage I – 78% (N=105). Stage II – 17% (N=23). Stage III – 0% (N=0). Stage IV – 5% (N=7). **Demographic:** Mean age at diagnosis (SD) [range] – 34.8 (8.9) [19 – 60}.	Convenience	67%.	N/A.	Cross sectional survey.	1.	**Semi structured interview:** Demographics (paired relation. Employment status, previous sever somatic disease or injury, previous mental problems, sleeping problems, satisfaction with information provided by local hospital). Interviewer determined if the TCP was “well informed”. **Medical records: h**istology, TC stage, time since diagnosis. **Questionnaires:** Trauma: Impact of Event Scale (IES). Anxiety and depression: Hospital Anxiety and Depression Scale. Mood affect: Positive and Negative Affect Scale (PANAS). Neuroticism: Eysenck Personality Questionnaire (EPQ). Hazardous alcohol use: The CAGE Questionnaire. Cognitive Function: Paired Associates Learning. Choice Reaction Time (CRT). Spatial Working Memory (SWM). Hopkins Verbal Learning Test Revised (HVLT-R). Grooved Pegboard. Trail Making Test A1B . Color-Word Interference Test (CW) 1–4. Word Fluency Test (FAS). Stockings of Cambridge (SOC) and/or Intra-Extra Dimensional Set Shifting (IED).
Smith et al., [18] Australia.	Explore the unmet supportive care needs of TCS.	14 – Metropolitan cancer centers (individual centers not identified).	N=224.	**Clinical:** Surveillance/surgery alone – 23% (N=55). Radiotherapy – 21% (N=52). Chemotherapy – 37% (N=90). **Demographics:** Not reported	Convenience	70% of contact able. 50% of eligible.	N/A.	Cross sectional survey.	1.	**Questionnaires:** Unmet supportive care needs: Cancer Survivors’ Unmet Needs (CaSUN). Psychological distress: Depression Anxiety Stress Scales short-form (DASS21). Health related quality of life: SF-36v2.
Smith et al., [23] Australia.	Determine the frequency and severity at which TCS experience psychological distress and health-related quality of life and identify correlates.	14 - Metropolitan cancer centers (individual centers not identified).	N=244.	**Clinical:** Surveillance/surgery alone – 23% (N=55). Radiotherapy – 21% (N=52). Chemotherapy – 37% (N=90). Chemotherapy + radiotherapy + further surgery – 19% (N=45) (RPLND – 67% (N= value not reported)). **Demographics:** Not reported	Convenience	70% of contact able. 50% of eligible.	N/A.	Cross sectional survey.	1.	**Questionnaires:** Psychological distress: Depression Anxiety Stress Scales-Short Form (DASS21). Generic Health Related Quality of Life: SF-36v2. Testicular Cancer Health Related Quality of Life European Organization for Research and Treatment of Cancer (EORTC) TC module QLQ-TC26.Coping style: Mental Adjustment to Cancer Scale (MAC). Unmet supportive care needs: Cancer Survivors’ Unmet Needs measure (CaSUN).
Soleimani et al., [51] Canada.	To identify the psychosocial needs of individuals with germ cell tumours	1 - B. C Cancer.	N=349.	**Clinical:** Pure seminoma: 55.9% (N=195). Mixed germ cell tumour: 22.1% (N=77). Other: 22.1% (N=77). **Demographics:** Median age (years) – 33.	Convenience	Not reported.	N/A.	Cross sectional survey.	1.	Psychological distress PsychoSocial Screen for CANcer. Other domains Revised (PSSCAN-R) and the Canadian Problem Checklist (CPC).
Stouten-Kemperman et al., [52] Netherlands	To determine the effects of chemotherapy on the brains and cognitive functioning of TCS.	1 - Netherlands Cancer Institute.	N=45.	**Clinical:** Chemotherapy/surgery only **Demographic:** Mean age (SD): 43.1 (7.5)/48.2 (9.5).	Convenience	55%.	N/A.	Casual comparative	1.	**Neurocognitive tests** Executive function: Controlled Oral Word Association Test Word Fluency, Trail Making Test card B, Tower of London, Mental rotation Task. Visual memory: Visual Reproduction Test of the Wechsler Memory Scale-Revised. Verbal memory: Dutch version of the California Verbal Learning Test. Motor speed: Finger-tapping. Processing speed: Digit Symbol-Coding Test of the WAIS-III. Trail Making Test card A. Attention: Eriksen Flanker Task. Digit Span of the WAIS-III **Questionnaires:** Quality of Life: European Organisation for Research and Treatment of Cancer (EORTC) Quality of Life Questionnaire-C30 (QLQ-C30). Perceived stress: Perceived Stress Scale. Trauma: Trauma Screening Questionnaire: Cognitive complaints: Cognitive Functioning Scale- Revised of the Medical Outcomes Study. Impact of Cancer: Impact of Cancer scale version 2 (IOCv2). Expectations of sterotyping: Stereotype vulnerability questionnaire. Perceived capability to work: Workability questionnaire. **General health:** Abdominal circumference. Systolic and diastolic blood pressure, **Brain imaging:** MRI
Tasdemir et al., [53] Turkey.	To determine the presence of sexual dysfunction, depression, anxiety, and gonadotropin hormone levels in TCS treated with chemotherapy.	Department of Medical Oncology and Urology, Inonu University Firat University.	N=27. Control size unknown.	**Clinical:** Not reported. **Demographics:** Mean age (SD) - 34 years (8.9).	Convenience	Not reported.	N/A.	Cross sectional survey.	1.	Depressive symptoms: Beck depression inventory-II (BDI-II) 22. Anxiety symptoms: Beck Anxiety Scale. Sexual Function: International Index of Erectile Function (IIEF-15). Blood samples: Serum Luteinizing Hormone. Follicle stimulating hormone. Testosterone.
Vehling et al., [54] Germany.	Explore TCS experience of positive and negative life changes after cancer	Uro-oncological outpatient ward, University Medical Center, the University Cancer Center Hamburg. Private practice, Hamburg, Germany.	N=164.	**Clinical:** Chemotherapy - 76% (N=124). Surgery – 98% (N=160). Radiotherapy – 23% (N=38). **Demographics:** Mean age (SD, range) – 44.4 (9.6, 24-77)	Convenience	61.1%.	N/A.	Cross sectional survey.	1.	Perceived positive and negative life changes: Modified Posttraumatic Growth Inventory (PTGI). Depression: Patient Health Questionnaire-9 (PHQ-9). Anxiety: Generalized Anxiety Disorder Screener-7 (GAD-7). Symptom burden: Memorial Symptom Assessment Scale—Short Form (MSAS-SF).
Vehling et al., [55] (same study as above) Germany.	Investigate and establish the presence of anxiety and depression in TCS and analyse the influence of correlates.	Uro-oncological outpatient ward, University Medical Center, University Cancer Center Hamburg . Private practice in Hamburg (no further detail supplied).	N=164.	**Clinical**: None – 2% (N=4). Orchiectomy only – 56% (N=92). Surgery more than orchiectomy – 42% (N=68). **Demographics:** Mean age (SD) – 44.4 (9.6).	Convenience	61.1%.	N/A.	Cross sectional study.	1.	Sociodemographic data: Unspecified ‘standardised self-report questionnaire’. Anxiety: Generalized Anxiety Disorder Screener-7 (GAD-7). Depression: Patient Health Questionnaire-9 (PHQ-9). Physical symptoms: The Memorial Symptom Assessment Scale – Short Form.
Wang and Hoyt, [24] USA.	To explore the relationship between benefit finding and psychological adjustment in TCS	Number unspecified. Men enrolled in the California Cancer Registry.	N=171.	**Clinical:** Surgery and chemotherapy. I – 35% (N= value not reported). II or III – 65% (N= value not reported). **Demographics:** Mean age (SD) – 25.2 (3.3).	Convenience	Not reported.	N/A.	Cross sectional survey.	1.	**Questionnaires:** Benefit from testicular cancer: The Benefit Finding Scale (BFS). Threat to masculinity: Cancer-related Masculine Threat Scale. Psychological Adjustment to cancer: The Positive and Negative Affect Schedule (PANAS). The Center for Epidemiologic Studies Depression Scale (CES-D). Self-esteem: Rosenberg Self-Esteem Scale.
Wefel et al., [56] USA.	To investigate the influence of chemotherapy treatment on cognitive functioning in TCS	Genito-urinary medical service of MD Anderson Cancer Centre, Houston, Texas.	N=69.	**Clinical:** all treated by chemotherapy. Stage I – 92.9% (N=13) Stage II – 7.1% (N=1) Stage III – 0% (N=0) **Demographics:** Mean age (SD) [range] - 31.0 (±7.5) [18.5 – 50.7].	Convenience	Not reported.	Unclear	Prospective longitudinal.	3.	**Neuropsychological tests:** Attention: WAIS-R Digit Span Psychomotor speed: WAIS-R Digit Symbol. Trail Making Test Part A. Language: MAE Controlled Oral Word Association. Learning and memory: HVLT Trials 1-3, Total Recall. Executive function: Trail Making Test B. Motor: Grooved Pegboard (dominant hand). Grooved pegboard (nondominant hand) **Questionnaires:** Depression: Centre for Epidemiologic Studies Depression Scale. Anxiety: State-Trait Anxiety Inventory – State score **Biological data:** Human chorionic gonadotropin. Alpha fetoprotein. Testosterone. Lactate dehydronase
Wefel et al., [57] (same study as above) USA.	To investigate the prevalence of cognitive impairment in TCS prior to the administration of adjuvant therapies.	Genito-urinary medical service of MD Anderson Cancer Centre, Houston, Texas.	N=69.	**Clinical:** Orchiectomy – 100%. I – 51% (N=35) II- 33% (N=23) III – 15% (N=10) **Demographic:** Mean age (SD) [range] - 31.0 (±7.5) [18.5 – 50.7].	Convenience	Not reported.	N/A.	Cross sectional.	1.	**Neuropsychological tests:** Attention: WAIS-R Digit Span. Psychomotor speed: WAIS-R Digit Symbol. Trail Making Test Part A. Language: MAE Controlled Oral Word Association. Learning and memory: HVLT Trials 1-3, Total Recall. Executive function: Trail Making Test B. Motor: Grooved Pegboard (dominant hand). Grooved pegboard (nondominant hand) **Questionnaires:** Depression: Centre for Epidemiologic Studies Depression Scale. Anxiety: State-Trait Anxiety Inventory – State score **Biological data:** Human chorionic gonadotropin. Alpha fetoprotein. Testosterone. Lactate dehydronase
Wibe et al., [34] Norway.	Determine If online patient nurse communication service meets patient needs and how patients utilised the service	Cancer department, Tertiary Hospital. (Hospital not specified).	Messages N=54. From 12 patients. 5 Interviews from 5 individuals included in study.	**Clinical:** Diagnosed with testicular cancer in the last three months and were undergoing treatment at the tertiary hospital – (100%). **Demographics:** Age (range): 24 – 51 years. Mean age: 37. Median age: 36.	Convenience	41.6% (for interviews).	N/A.	Phenomenological study.	1.	Semi-structured Interviews. Data from online patient-nurse communication.
Wortel et al., [58] Netherlands	To determine the effect of Radiotherapy and Orchiectomy on the sexual functioning and body image of TCP.	1 – Department of Radiation Oncology. Further details not reported.	N=120.	**Clinical:** combination therapies. pT1 – 114 (71). pT2 – 47 (29). N0 – 145 (90). N1 – 14 (9). N2 – 2 (1). **Demographic:** Median age at treatment (range): 36 (18-70).	Convenience	68%.	25%	Prospective longitudinal.	3.	**Questionnaire:** Dutch translation of sexual function questionnaire.

Cognitive Impairment (CI); testicular cancer (TC); testicular cancer patient (TCP); testicular cancer survivor (TCS); bilateral testicular cancer (BTC); contralateral germ cell neoplasia (GCNIS); quality of life (QoL); unilateral testicular cancer (UTC); contralateral germ cell neoplasia in situ (cGCNIS); retroperitoneal lymph node dissection (RPLND); Brief Male Sexual Function Inventory for Urology (BMSFIU); Leisure time physical activity; three cycles of bleomycin, etoposide, and cisplatin (BEPX3); four cycles of etoposide and cisplatin (EPX4); 4 cycles of bleomycin, etoposide and cisplatin (BEPX4); 4 cycles of etoposide, ifosfamide, and cisplatin (VIPX4); 5 cycles of etoposide, ifosfamide, and cisplatin (VIPX5); week (wk); metabolic equivalent of task (METs); International Germ Cell Cancer Collaborative Group (IGCCCG); bleomycin, etoposide and cisplatin (BEP); testicular cancer survivor (TCS); fear of recurrence (FoR); Beck Depression Index (BDI); benefit finding (BF); pT1; pT2;N0; N1; N2; scale for chemotherapy induced long term neurotoxicity (SCIN); NART; bleomycin, etoposide, cisplatin (BEP); post chemotherapy retroperitoneal lymph node dissection (PC-RPLND); interquartile range (IQR); low limit of normal (LLN); upper limit of normal (ULN); follicle stimulating hormone (FSH); luteinizing hormone (LH); hospital depression and anxiety scale (HADS)

**Table 3:**
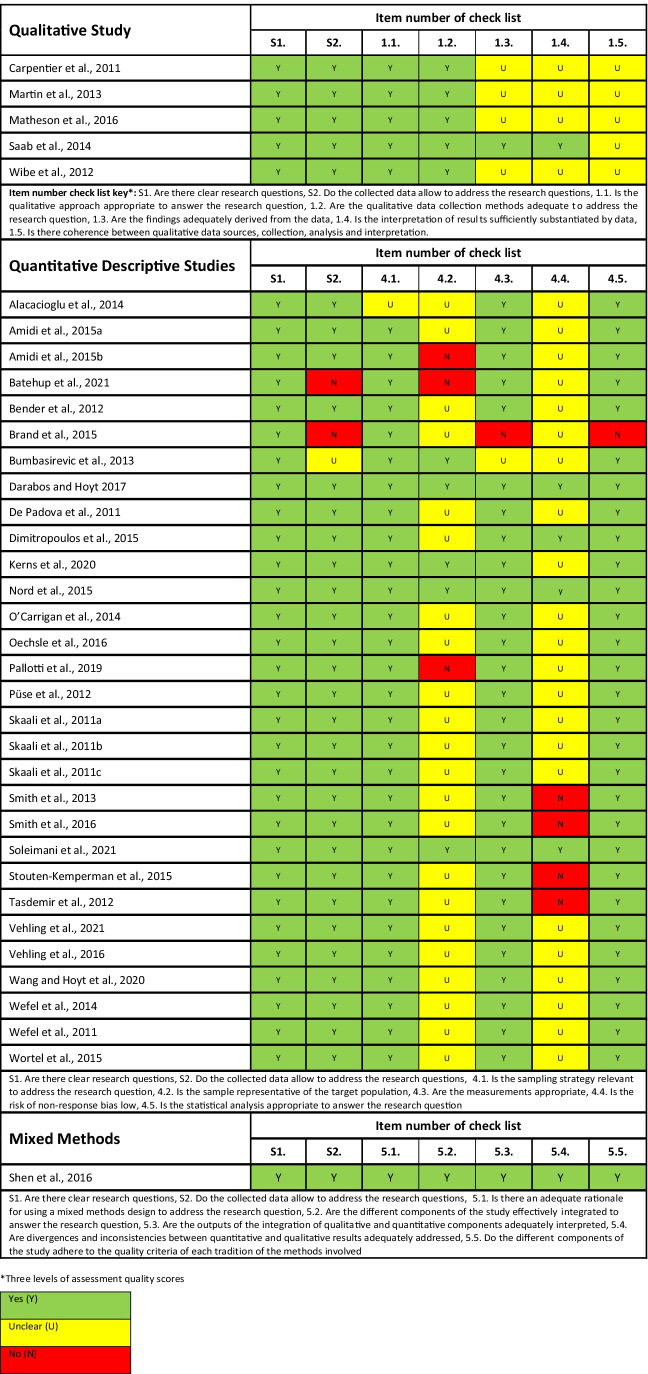
Quality appraisal of primary studies

### Frequency of unmet supportive care needs

The frequency of unmet supportive care needs varied within and between studies (see Table 4). In descending order of frequency of need, psychological needs were identified in 26/36, physical needs 18/36, interpersonal/intimacy needs 19/36, health system/information needs 11/36, cognitive needs 9/36, social needs 7/36, and of equal frequencies (4/36) for family, practical, and patient-clinician information needs. Only one study explored spiritual needs [51], and no daily living needs were identified.✔

**Table 4:** Frequency of unmet needs by domain

Study	Physical Needs	Psychological/ Emotional Needs	Cognitive Needs	Patient-Clinician communication	Health System/ Information Needs	Spiritual Needs	Daily Living Needs	Interpersonal/ Intimacy Needs	Practical Needs	Family Related Needs	Social needs	Number of domains explored within each study
Alacacioglu et al., 2014 [22]	✔	✔	-	-	-	-	-	✔	-	-	-	3
Amidi et al., 2015a [35]	-	✔	✔	-	-	-	-	-	-	-	-	2
Amidi et al., 2015b [36]	-	✔	✔	-	-	-	-	-	-	-	-	2
Batehup et al., 2021 [37]	-	✔	-	✔	✔	-	-	✔	-	✔	✔	6
Bender et al., 2012 [15]	✔	✔	-	-	✔	-	-	✔	✔	-	✔	6
Brand et al., 2015 [38]	✔	✔	-	-	✔	-	-	✔	-	✔	-	4
Bumbasirevic et al., 2013 [39]	✔	✔	-	-	-	-	-	✔	-	-	-	3
Carpentier et al., 2011 [31]	-	✔	-	-	-	-	-	✔	-	-	✔	3
Darabos and Hoyt, 2017 [40]	-	✔	-	-	-	-	-	-	-	-	-	1
DePadova et al., 2011 [41]	✔	✔	-	✔	✔	✔	✔	✔	✔	✔	✔	8
Dimitropoulos et al., 2016 [42]	-	-	-	-	-	-	-	✔	-	-	-	1
Kerns et al., 2020 [43]	✔	✔	-	-	-	-	-	✔	✔	-	-	4
Martin et al., 2013 [21]	-	-	-	-	✔	-	-	-	-	-	-	1
Matherson et al., 2016	✔	✔	-	-	✔	-	-	✔	-	-	✔	5
Nord et al., 2015 [44]	-	-	-	-	-	-	-	-	✔	✔	-	1
O’Carrigan et al., 2014 [45]	✔	✔	-	-	-	-	-	-	-	-	-	2
Oechsle et al., 2016 [20]	✔	✔	✔	-	-	-	-	✔	-	-	-	4
Pallotti et al., 2019 [46]	✔	-	-	-	-	-	-	✔	-	-	-	2
Pühse et al., 2012 [47]	✔	-	-	-	-	-	-	✔	-	-	-	2
Saab et al., 2014 [33]	-	✔	-	-	-	-	-	✔	-	-	-	2
Shen et al., 2016 [59]	-	✔	-	✔	✔	-	-	-	-	-	-	3
Skaali et al., 2011a [48]	-	-	✔	-	-	-	-	-	-	-	-	1
Skaali et al., 2011b [49]	✔	-	✔	-	-	-	-	-	-	-	-	2
Skaali et al., 2011c [50]	-	✔	✔	-	-	-	-	-	-	-	-	2
Smith et al., 2013 [18]	-	✔	-	✔	✔	-	-	✔	✔	-	✔	6
Smith et al., 2016 [23]	✔	✔	-	-	-	-	-	✔	-	-	✔	4
Soleimani et al., 2021 [51]	✔	✔	-	-	✔	✔	-	-	✔	✔	-	6
Stouten-Kemperman et al., 2015 [52]	✔	✔	✔	-	-	-	-	-	✔	-	-	4
Tasdemir et al., 2012 [53]	-	✔	-	-	-	-	-	✔	-	-	-	2
Vehling et al., 2021 [54]	✔	✔	-	-	-	-	-	-	-	-	-	2
Vehling et al., 2016 [55]	✔	✔	-	-	-	-	-	-	-	-	-	2
Wang and Hoyt, 2020 [24]	-	✔	-	-	-	-	-	-	-	-	-	1
Wefel et al., 2014 [56]	-	-	✔	-	-	-	-	-	-	-	-	1
Wefel et al., 2011 [57]	-	✔	✔	-	-	-	-	-	-	-	-	2
Wibe et al., 2012 [34]	-	-	-	-	✔	-	-	✔	-	-	-	2
Wortel et al., 2015 [58]	✔	✔	-	-	✔	-	-	✔	-	-	-	4
Number of domains explored across all studies	18	26	9	4	11	1	0	19	4	4	7	

### Unmet supportive care needs by domain

#### Psychological/emotional needs

Individuals affected by testicular cancer commonly reported unmet psychological/emotional needs. Stress [15, 23, 36, 37], anxiety [23, 43, 45, 51, 53, 54, 57], depression [23, 39, 43, 45, 54], fear of recurrence [23, 37, 38, 41, 59], and body image issues [15, 32, 37, 38, 52, 58] were commonly experienced. Timely intervention for emotional support [23, 32, 37, 59], coping with threats to masculinity [23, 24, 31] and counselling for issues about infertility were needed [32, 33]. Men were embarrassed to disclose concerns about the signs and symptoms of testicular cancer [31], sexual functioning [34], and apprehension to share their diagnosis to the people in their lives [21, 33]. Other challenges included their own self-regulation of managing their own expectations of being a “cancer survivor” [15, 23, 37], how to move on with their lives [15, 23, 37], a lack of emotional support [15, 23], and sadness [20, 32]. Overall, studies reported negative impacts on mental health, reduced emotional functioning, low mental component summary scores [23], and reduced emotional vitality [39].

“…the bounce back from this was something that I couldn't cope with emotionally because I've never really dealt with a lot of emotions … I'm a guy … you need to be strong and that's what I was taught and you just deal with it and suck it up …” (page e16) [59]

#### Physical needs

Across the studies, there were a range of physical needs which required self-management support from healthcare professionals. Commonly, testicular cancer survivors experienced fatigue [20, 39, 41, 59], lack of energy [20, 54], drowsiness [20, 54], pain [20, 23, 43], hair loss [15, 20], and sleep disturbances [20, 54]. Men grappled with chemotherapy-induced alopecia and reported needing help with hair loss, but was not provided with any support or education on preventative strategies, such as scalp-cooling [15].

“Losing my hair was probably more devastating than losing my testicle I think. Because I went from liking my hair to having none in about three days. And that was a big adjustment. Even though a lot of guys you see on the street shave their head, and have short hair when it first happened midway through the chemotherapy … I hadn’t shaved in three or four days or whatever, it was just falling off. That was probably as devastating as anything, for me. It was just like, ‘wow’ (Participant 1)” (page 742) [31].

Other less commonly experience symptoms but still caused distress among testicular cancer survivors included itching, cough, sweats, shortness of breath, dizziness, skin changes, mucositis, numbness and tingling, feeling bloated, changed taste, urination difficulties, diarrhoea, and constipation [20]. Additionally, there were problems with fertility [23, 38, 41, 58], hypogonadism [43, 45, 46], higher white matter hyperintensities and radial kurtosis [52], and low testosterone [38] were reported. Chemotherapy-induced consequences such as obesity, peripheral sensory neuropathy, renal disease, tinnitus, hearing loss, Raynaud phenomenon, and autonomic neuropathy were frequently experienced. However, long-term conditions such as hypertension, thromboembolism, hypocholesteraemia, peripheral artery disease, diabetes, thyroid disease, coronary artery disease, transient ischaemic attack, and stroke were identified less frequently in this young population [43]. Testicular cancer survivors were found to have lower mean vitality, physical functioning, physical role functioning, and general health when compared to the general healthy population [23]. Men had physical concerns related to having one testicle which was intertwined with psychological consequences and intimacy concerns [38]. Noteworthy, 20% of the participants who received a prothesis were unhappy with the aesthetic result [18].

#### Interpersonal/intimacy needs

Individuals affected by testicular cancer reported needing help with their sex life [18, 37] because they were embarrassed to discuss this with healthcare professionals [38]. Only 14% of testicular cancer survivors reported having “none/a little” issues in their sex life [41]. Some men reported a decreased level of sexual function post-treatment and long-lasting into survivorship [39]. The most commonly experienced issues for these young men included erectile dysfunction [39, 43, 46, 47, 53], reduced erectile rigidity [58], and inability to maintain an erection during intercourse due to chronic pain [47]. Other concerns were reduced sexual interest [20, 23, 59], lack of sexual desire [39, 46, 47], and in frequent of sexual activity [22, 47, 58]. Disorders of ejaculation were prevalent [39, 42, 47] with loss of antegrade ejaculation [42]. Decreased sexual satisfaction [47], enjoyment of intercourse [42], intercourse satisfaction [46], reduced pleasure [58], decreased orgasm frequency [42], and decreased orgasm intensity [47] were also reported. Problems within intimate relationships also surfaced because some participants felt they could not speak to their partner about sexual issues [23], which reported decreased general satisfaction [46] or decreased satisfaction with their sexual life and relationship [42]. For other young men, they reported concerns about finding a future partner [38] and did not know how to communicate to discuss this sensitive issue with partners or healthcare professionals [22]. Treatments including chemotherapy and extended lymph node dissection were associated with poor sexual functioning [42, 43].

“Sexual questions for example, which might have come up during the doctors’ rounds … This might be easier to ask about in an e-mail to a person that you don’t know than when the doctor asks: “What about your … (sexual function)?” Then you answer: “Oh, that’s OK” or “That’s normal” or whatever …” (page 4) [34].

#### Health system/information needs

Some studies identified that men wanted improved communication in the healthcare system to address problems with co-ordination of their care [23, 37]. However, participants also needed informational support to provide reassurance that they were receiving the best care [23, 37] and that their complaints were being addressed in a timely manner. Informational supports within the healthcare system were, at times, inadequate for patients [15, 59] and their partners [59] and omitted recovery expectation post-treatment [15, 59] to inform rehabilitation care plans.

“There was no discussion that I remember that was any, you know, ‘if you're feeling like this, then come and talk to us,’ or, you know, ‘there's counselling available,’ or anything like that. I don't recall anything like that for the psychological side of any concerns, really.” (page 16) [59].

It was important that men received understandable and up-to-date information to support decision-making [37] at diagnosis and treatment phases [51]. Fundamental gaps in information provision were observed for knowledge and understanding of which treatments men received, and associated risks of treatments, lifestyle advice to support self-management within the multidisciplinary team, and timely access to results, and how to self-report concerning symptoms to healthcare professionals [59]. Patients also identified that websites were critical for accessing information [41] but raised questions about the quality of information which is being accessed by men.

Men reported informational needs related to deciding on which prothesis to proceed with [15], and 44% of patients did not receive any information related to the option of a prothesis [58]. Men also wanted information in relation to how to access to complementary or alternative therapies [15] and information in relation to sexual recovery [38]. Noteworthy, 50% of testicular cancer survivors did not know what information supports were available to them [59].

#### Cognitive needs

Testicular cancer survivors frequently experienced cognitive impairment [20, 35, 36, 48–50, 52, 56, 57] or cognitive decline over time [48, 56] post-treatment and into survivorship. Participants self-reported cognitive difficulties [20, 50, 52], but were not always reflected in objective neuropsychological testing used to evaluate cognitive decline [49]. One study [20] found that of the 32% of participants who reported difficulty concentrating, 8% of the participants found it highly distressing. None of the participants across these studies reported receiving timely intervention or support for their difficulties with cognition. Evidence identified that as many as 58% of testicular cancer survivors can experience cognitive impairment [35, 36] which is significant given this young cohort of men who could be either studying or working in paid employment. One study [49] found that self-reported cognitive impairment was associated with psychological distress.

‘It just feels kind of like you’re incomplete. Just as a person you feel like you’re missing something you’re supposed to have. I guess it’s just the fact that it doesn’t have any real effect but there’s still something missing. So it’s just that weird dichotomy’ (Participant 9)” (page 742) [31].

#### Social needs

Men diagnosed with testicular cancer reported the need to talk to other survivors [15, 23, 37]. Other social needs included how to navigate sensitive conversations of their cancer diagnosis in the work environment [15, 37]. Testicular cancer survivors and their caregivers indicated that cancer made their social relationships difficult [41] and they wanted help and advice in how to create new relationships with intimate partners [37]. Testicular cancer survivors needed help knowing how to deal with this impact on relationships [37] and were found to have lower social functioning than healthy populations [23].

“‘I just think it just helped just reassure me, like I wasn’t a nutter, or some weirdo, and you’re not the only person, you won’t ever be the only person who’s gone through it’ (P20, T1, 22 years., single, surgery and chemotherapy)” (page 199) [32].

#### Family-related needs

Young men expressed needs in knowing how to support their partners or families [37], how to communicate with their young children [38], and concerns about being unable to have children due to fertility issues [38]. It was common for men to experience issues within their existing relationships which caused emotional strain [41, 51].

“An infertile man … the way people perceive him makes him want to beat himself … I suffered … a man is about sex and kids to a certain extent.” (page 206) [33].

#### Practical needs

Practical unmet needs included a lack of assistance to access government benefits [15, 23], guidance on life insurance, and accessible parking at the hospital [23]. Testicular cancer survivors reported having difficulties with their work or study [41], experienced higher unemployment than general populations [43], with an increased risk of loss of employment [44]. One study [52] highlighted that upon testicular cancer survivors return to work, some required changes to their workplace to return to work, and others did not return to their previous role because of cognitive impairment [52].

“I don’t know what to look for, I don’t know what to expect.” (page e16)[59].

#### Patient-clinician communication needs

These were some important implications for patient-clinician communication needs identified across four studies [18, 37, 41, 59]. Men expressed that they wanted to feel more supported in the self-management of their health in partnership with their healthcare team [23, 37].

“There was no discussion that I remember that was any, you know, ‘if you're feeling like this, then come and talk to us,’ or, you know, ‘there's counselling available,’ or anything like that. I don't recall anything like that for the psychological side of any concerns, really. The attitude seemed to be, if something bothers you, tell us and we'll deal with it. We're not going to tell you in advance what any of those things might be.” (page e16) [59].

However, on the whole, men expressed satisfaction and confidence with their patient-clinician communication needs [59].

#### Spiritual needs

Only two studies [37, 51] explored the spiritual needs in this patient group. One study [51] identified that faith and the meaning of life were rated as least concerning unmet needs [43] and testicular cancer survivors reported that they had no unmet spiritual needs [37] in this young patient group.

#### Daily living needs

Across all the studies, no information was reported in relation to daily living unmet needs of individuals affected by testicular cancer.

## Discussion

This systematic review sets out to identify the unmet supportive care needs among young men diagnosed with testicular cancer. The included studies identified that needs varied in terms of distress and frequency across different domains of supportive care. The current review found emotional, intimacy, and physical needs to be the most frequently reported unmet domains of care. This is a similar outcome to reviews conducted in other cancer populations [7–9, 12]. However, cognitive needs of individuals affected by testicular cancer were problematic for these young men, compared to mainstay experiences of unmet needs in other cancer groups [7–9, 12]. Cognitive impacts were investigated at varying time points from immediately post-orchiectomy [35, 48–50, 56, 57] and into survivorship [20, 35, 52]. Cognition was found to be negatively impacted by orchiectomy alone [35, 57]. However, evidence about the relationship with chemotherapy on cognitive function [49, 52, 56] or indeed lack of association [35, 48] is conflicting. Amidi [35] found that cortisol levels were associated with impaired cognitive function, while increased C-reactive protein was associated with poor verbal fluency test outcomes. Furthermore, self-reported cognitive problems were correlated to Raynaud-like symptoms and fatigue [49], and cognitive decline was also associated with hearing loss [48]. Self-reported measures of cognitive impairment have also been linked to emotional/psychological needs [49, 50]. Therefore, it should be noted that self-reported cognitive issues, and objective measurable cognitive impairment in testicular cancer survivors is conflicting [49] which is consistent within the literature in other cancer populations [60]. Furthermore, a meta-analysis [61] of the effects of chemotherapy on cognition in patients with cancer remains unclear. Given the findings from this current review, future studies should explore mechanism pathways for both objective, and subjective measures in relation to cognitive impairment in this patient group. Gaining this information will help to leverage the development of interventions for cognitive pre -and/or rehabilitation.

This review found hypogonadism to occur in testicular cancer survivors. Hypogonadism is known to result in low testosterone in the male sex [62]. Low testosterone has been associated with worse sexual functioning [63]. This review identified that hypogonadism was reported, and one study found that it was not related to sexual functioning [46]. It is established that sexual dysfunction can be of psychogenic nature [63] and the findings of this review support that this may be true for some testicular cancer survivors. One study [58] found that in men who received a prothesis they reported no sexual dysfunction, whereas men who did not have a prothesis did self-report issues with sexual dysfunction. Body image concerns have been found to be associated with sexual dysfunction in testicular cancer survivors [64]. Intimacy needs are high in patient populations where the cancer affects the reproductive organs or secondary sexual characteristics [65] which can negatively impact the sexuality of the affected person. However, it is unlikely all sexual dysfunction reported in this review is only attributed to a psychogenic nature given the consistently high rates of sexual dysfunction in the testicular cancer survivor populations [66]. Other issues encountered by these men were a lack of opportunity to discuss these problems with their intimate partner or healthcare professions, often because of embarrassment. Therefore, healthcare professionals should be mindful of these concerns and tactfully and sensitively explore concerns to ensure that men receive timely intervention.

Health system/information needs were frequently unmet. Reasons for these gaps were not explored in the included studies but would be an important clinical focus for future research. The spiritual needs of testicular cancer survivors were rarely discussed, and one study [37] found that testicular cancer survivors did not report any unmet spiritual needs. It is unclear to determine the spiritual needs of individuals affected by testicular cancer because of the lack of data. It is also noteworthy that the men represented in this systematic review also did not express concerns with existential issues, or fear of death and dying, concerns commonly experienced in other cancer populations [7–9, 12]. It would be important to explore whether these were concealed concerns because of their age or reticence to disclose, but ultimately this remains unknown. Likewise, there were no identified daily living needs which might be explained in part because of the young age demographic, but men did share challenges about their practical needs.

Testicular cancer survivors were found to experience greater work loss and take more sick leave than the general population [44]. Unemployment rates for testicular cancer survivors were also higher than the general population [43]. Furthermore, peripheral neuropathy was associated with unemployment and disability leave [43], and receiving four or more courses of chemotherapy was associated with work loss [44]. There are practical needs which have also been identified in the wider cancer care literature [67, 68]. However, specific to this young population were concerns about work, school, and finances compared to older patients [51]. This is logical as this population is still generally establishing a career and financial independence [69]. Family-related needs were infrequently investigated in the literature. The family needs of individuals affected by testicular cancer should be a central focus for future research, particularly given the impact on intimacy and relationships.

Lastly, future directions for research should focus on developing a core outcome set (COS) for testicular cancer survivorship research. This review has identified significant heterogeneity of study outcomes and in particular patient reported outcomes measures (PROMs). There were a total of 57 different PROMs used across the studies in this systematic review and a range of diverse methods used.

### Limitations

This systematic review has many strengths including the clear and specific methodology which followed a registered priori protocol. In addition, to the independent reviewer’s contributions throughout the entirety of the systematic review process, the study provided insights across heterogenous study populations in terms experiences of unmet supportive care needs. One of the major challenges of this review was combining heterogeneous methodologies, and our findings are constrained due to the methodological limitations of the studies included. The review only included articles in the English language, and as such, it may limit our understanding of the area globally considering cultural and societal differences. The review also did not include any participants from low- to middle-income countries, and efforts/funding should be targeted to support cancer care in developing nations as a future priority. Lastly, this review only included studies published in the English language, and therefore by omission, valuable insights may have been missed.

## Conclusion and implications for cancer survivors

The interrelated nature of the unmet supportive care needs experienced by individuals affected by testicular cancer emphasises the importance of holistic, person-centred approaches to care delivery. The contemporary evidence identified in this review highlights areas of clinical practice that require improvement to enhance the healthcare experiences of individuals affected by testicular cancer.

### Supplementary Information

Below is the link to the electronic supplementary material.

Supplementary file1 (DOCX 17 KB)

Supplementary file2 (DOCX 17 KB)

Supplementary file3 (DOCX 44 KB)

Supplementary file4 (DOCX 40 KB)
