# Supplementary material for: Identifying the unmet supportive care needs of individuals affected by testicular cancer: a systematic review
Source: J Cancer Surviv. 2022 Jul 4;18(2):263–87. doi: 10.1007/s11764-022-01219-7 (PMC10960773; doi:10.1007/s11764-022-01219-7)
Supplement: Supplementary file 2 — Supplementary file2 (DOCX 17 KB) [file 11764_2022_1219_MOESM2_ESM.docx]

**Supplementary Table 2 - Record of database searches**

| Database: APA PsycINFO | | | |
| --- | --- | --- | --- |
| Date of Search: 17/11/2021 | | | |
| Symbols used in this document: | | | |
| “ ” finds a phrase | | | |
| Asterisk (*) = truncates stem of a word | | | |
| **Search #** | **Concept/Explanation** | **Search Terms/Strategy** | **# of Results** |
| #1 | “Population” – individuals affected by testicular cancer | "Testicular Neoplasm*" OR "testicular cancer*" | 320 |
| #2 | “Outcomes of interest” | "unmet supportive care needs" OR "patient needs" OR "care needs" OR "family needs" OR "caregiver needs" OR "supportive care" OR "supportive care needs" OR "health system" OR "health information" OR "patient-clinician communication" OR "patient care team" OR "multidisciplinary care team" OR "patient care needs" OR "patient comfort" OR "patient care planning" OR "patient care bundles" OR "patient care" OR "patient centered care" OR "person centered care" OR "family centered care" OR "progressive patient care" OR "continuity of patient care and oncology and palliative and primary care" OR "continuity of patient care" OR "respite care" OR "social support" OR "social relationships" OR "support" OR "financial support" OR "nutritional support" OR "decision support techniques" OR "subacute care" OR "ambulatory care" OR "outpatients" OR "primary health care" OR "acute care" OR "inpatients" OR "hospital care" OR "health planning support" OR "behavioural symptoms" OR "symptom assessment" OR "symptom management" OR "urinary symptoms" OR "signs and symptoms" OR "symptom distress" OR "symptoms" OR "sexual dysfunction" OR "sexual health" OR "information literacy" OR "information needs" OR "needs assessment" OR "spiritual therapies" OR "spirituality" OR "religion" OR "faith" OR "belied system" OR "spiritual needs" OR "hospice and palliative care nursing" OR "palliative care" OR "pain" OR "pain management" or "pain measurement" OR "human needs" OR "physical needs" OR "emotional support" OR "emotional needs" OR "family support" OR "family involvement" OR "family engagement" OR "family needs" OR "social needs" OR "interpersonal relations" OR "interpersonal" OR "activities of daily living" OR "daily living needs" OR "bereavement" OR "grief" OR "loss" | 1,451,825 |
| #3 | “Outcome of interest and individuals affected by testicular cancer” | 1 AND 2  Limiters – English language and Peer Reviewed | 138 |

| Database: Cumulative Index to Nursing and Allied Health Literature (CINAHL) | | | |
| --- | --- | --- | --- |
| Date of Search: 17/11/2021 | | | |
| Symbols used in this document: | | | |
| MH = Main Heading or “CINAHL Heading” | | | |
| “ ” finds a phrase | | | |
| Asterisk (*) = truncates stem of a word | | | |
| **Search #** | **Concept/Explanation** | **Search Terms/Strategy** | **# of Results** |
| #1 | “Population” – individuals affected by testicular cancer | (MH "Testicular Neoplasms") OR “testicular cancer*” OR “testicular neoplasm*” | 3,537 |
| #2 | “Outcomes of interest” | "unmet supportive care needs" OR "patient needs" OR "care needs" OR "family needs" OR "caregiver needs" OR "supportive care" OR “health system” OR (MH “health information”) OR "patient-clinician communication" OR “patient care team” OR (MH "Multidisciplinary Care Team”) OR “patient care needs” OR “patient comfort” OR “patient care planning” OR “patient care bundles” OR (MH "Patient Care") OR (MH "Patient Centered Care") OR “person centered care” OR (MH "Family Centered Care") OR (MH "Progressive Patient Care") OR  "continuity of patient care and oncology and palliative and primary care" OR (MH "Continuity of Patient Care") OR (MH "Respite Care") OR “social support” OR  (MH "Support, Psychosocial") OR “social relationships” OR (MH "Financial Support") OR (MH "Nutritional Support") OR (MH "Decision Support Techniques") OR (MH "Subacute Care") OR (MH "Ambulatory Care") OR (MH "Outpatients") OR (MH "Primary Health Care") OR (MH "Acute Care") OR (MH "Inpatients") OR  “hospital care” OR “health planning support” OR (MH "Behavioral Symptoms") OR  “symptom assessment” OR “symptom management” OR “urinary symptoms” OR  (MH "Signs and Symptoms") OR "Signs and Symptoms" OR (MH "Signs and Symptoms, Digestive") OR (MH "Symptom Distress") OR (MH "Symptoms") OR  (MH "Sexual and Gender Disorders") OR “sexual dysfunction” OR (MH "Sexual Health") OR (MH "Information Literacy") OR "Information Literacy" OR (MH "Information Needs") OR “information needs” OR (MH "Needs Assessment") OR  "spiritual therapies" OR (MH "Spirituality") OR (MH "Religion and Religions") OR  "faith" OR “belief system” OR “spiritual needs” OR (MH "Hospice and Palliative Nursing") OR (MH "Palliative Care") OR (MH "Pain") OR (MH "Pain Measurement") OR (MH "Pain Management") OR (MH "Human Needs (Physiology) OR “physical needs” OR "emotional support" OR "emotional needs" OR "family support" OR “family involvement” OR “family engagement” OR “family needs” OR “social needs” OR (MH "Interpersonal Relations") OR “interpersonal” OR (MH "Activities of Daily Living") OR “daily living needs” OR (MH "Bereavement") OR (MH "Grief") OR (MH "Personal Loss") | 767,048 |
| #3 | “Outcome of interest and individuals affected by testicular cancer” | 1 AND 2  Limiters – English language and Peer Reviewed | 172 |

| Database: MEDLINE | | | |
| --- | --- | --- | --- |
| Date of Search: Date of Search: 17/11/2021 | | | |
| Symbols used in this document: | | | |
| MH = Main Heading or “MeSH term” | | | |
| “ ” finds a phrase | | | |
| Asterisk (*) = truncates stem of a word | | | |
| **Search #** | **Concept/Explanation** | **Search Terms/Strategy** | **# of Results** |
| #1 | “Population” – individuals affected by testicular cancer | “testicular cancer*" OR “testicular neoplasm*” OR (MH "Testicular Neoplasms") | 27,282 |
| #2 | “Outcomes of interest” | "unmet supportive care needs" OR "patient needs" OR "care needs" OR "family needs" OR  "caregiver needs" OR "supportive care" OR "supportive care needs" OR "heath system" OR  "health information" OR "patient-clinician communication" OR "cognitive needs" OR  (MH "Patient Care Team") OR (MH "Patient Comfort") OR "patient care needs" OR  "multidisciplinary care team" OR (MH "Patient Care Planning") OR (MH "Patient Care Team") OR (MH "Patient Care Bundles") OR (MH "Patient Care") OR (MH "Patient-Centered Care") OR "person centered care" OR "family centered care" OR (MH "Progressive Patient Care") OR "continuity of patient care and oncology and palliative and primary care" OR (MH "Continuity of Patient Care") OR (MH "Respite Care") OR  (MH "Social Support") OR "social networks" OR "social relationships" OR "support" OR  (MH "Financial Support") OR (MH "Nutritional Support") OR (MH "Decision Support Techniques") OR (MH "Subacute Care") OR (MH "Ambulatory Care") OR  (MH "Outpatients") OR (MH "Primary Health Care") OR "acute care" OR (MH "Inpatients") OR "hospital care" OR (MH "Health Planning Support") OR "behavioural symptoms" OR (MH "Symptom Assessment") OR "symptom management" OR  "urinary symptoms" OR (MH "Signs and Symptoms") OR "Signs and Symptoms" OR (MH "Signs and Symptoms, Digestive") OR "Symptom Distress" OR "symptoms" OR (MH "Sexual Dysfunction, Physiological") OR (MH "Sexual Dysfunctions, Psychological") OR (MH "Sexual Health") OR "Sexual Dysfunction" OR (MH "Information Literacy") OR "information literacy" OR "information needs" OR (MH "Needs Assessment") OR  (MH "Spiritual Therapies") OR (MH "Spirituality") OR (MH "Religion") OR "faith" OR "belief system" OR "spiritual needs" OR (MH "Hospice and Palliative Care Nursing") OR  (MH "Palliative Care") OR (MH "Pain") OR (MH "Pain Measurement") OR (MH "Pain Management") OR "Human Needs" OR "Human Needs (Physiology)" OR "physical needs" OR "emotional support" OR "emotional needs" OR "family support" OR "family inclusion" OR "family involvement" OR "family engagement" OR "family needs" OR "social needs" OR (MH "Interpersonal Relations") OR "interpersonal" OR "practical needs" OR "psychological assessment" OR (MH "Activities of Daily Living") OR "daily living needs" OR (MH "Bereavement") OR (MH "Grief") OR "loss" | 3,918,418 |
| #3 | “Outcome of interest and individuals affected by testicular cancer” | 1 AND 2  Limiters – English language and Peer Reviewed | 2,061 |
