# Supplementary material for: Identifying the unmet supportive care needs of individuals affected by testicular cancer: a systematic review
Source: J Cancer Surviv. 2022 Jul 4;18(2):263–87. doi: 10.1007/s11764-022-01219-7 (PMC10960773; doi:10.1007/s11764-022-01219-7)
Supplement: Supplementary file 3 — Supplementary file3 (DOCX 44 KB) [file 11764_2022_1219_MOESM3_ESM.docx]

**Table 8. Quantitative unmet supportive care needs**

| **Author and Year** | **Physical Needs** | **Psychological/Emotional Needs** | **Cognitive Needs** | **Patient-Clinician Communication Needs** | **Health System/Information Needs** | **Spiritual Needs** | **Daily Living Needs** | **Interpersonal/intimacy Needs** | **Practical Needs** | **Family Related Needs** | **Social Needs** |  |
| --- | --- | --- | --- | --- | --- | --- | --- | --- | --- | --- | --- | --- |
| Alacacioglu et al., 2014. | TCSs’ physical functioning was lower than healthy controls. | There was not any statistically significant difference between the TCSs and healthy men, and the depression and anxiety rates of TCSs were lower than the control group. | Not reported. | Not reported. | Not reported. | Not reported. | Not reported. | TCS had higher sexual dissatisfaction scores compared to healthy controls.  There was a reduction in sexual functioning of the TCSs from 50 to 30 %.  The effects of depression were associated with satisfaction, avoidance, touch, and erectile dysfunction. | Not reported. | Not reported. | Not reported. | |
| Amidi et al., 2015a. | Not reported. | TCS had higher levels of stress than healthy controls (p<0.001). | Overall cognitive performance in TCS (M= -0.42, SD = -0.6) was lower than healthy controls (M= -0.01, SD = 0.6), (t(79) = -2.9, p= 0.004). Cognitive impairment was present in 58% of testicular cancer survivors and 24% of healthy controls (χ2 (1) = 8.9, p= 0.004).  Impact of events scale – revised negatively associated with overall cognitive function (p = 0.04)  Cortisol levels were associated with worse outcomes in 3/6 neuropsychological outcomes.  C-reactive protein was associated with verbal fluency test outcomes (p = 0.05). | Not reported. | Not reported. | Not reported. | Not reported. | Not reported. | Not reported. | Not reported. | Not reported. | |
| **Amidi et al., 2015b.** | Not reported. | Mean perceived stress (PSS) (SD): 14.6 (6.6) [4 – 39}.  Mean depressive symptoms (BDI-II) (SD) [range] – 6.4 (7.0) [0 - 39}. | 62.5% of TCS were cognitively impaired (N=45/72).  CI was observed in multiple outcomes related to verbal learning and memory (29 to 33 % of participants), visual learning and memory (14–28 %), processing speed (8–24 %), executive functioning (17 %), and attention and working memory (4–15 %). No association was found between treatment modality (surgery ± chemotherapy) and CI. | Not reported. | Not reported. | Not reported. | Not reported. | Not reported. | Not reported. | Not reported. | Not reported. | |
| Batehup et al., 2021.  Mean number of unmet needs at T0 (SD) -2.01 (3.12)  Mean number of unmet needs at T2 (SD) - 1.76 (3.88) | Not reported. | ‘Help to cope with my concerns that my cancer will recur’ - 22% (N=9)/14.6% (N=6).  ‘Help to reduce stress in my life” – 15%(N=6)/12.5%(N=5).  ‘To help move on with my life” – 7.3%(N=3)/ 10%(N=4).  ‘For others to acknowledge the impact of cancer on my life’ – 5.1%(N=2)/5.1% (N=2).  ‘Emotional support for me’ – 9.8%(N=4)/9.8%(N=4).  ‘Help to make decisions in uncertain times’ – 14.6%(N=6)/ 7.5% (N=3).  ‘Help to make my life count’ – 4.9%(N=2)/7.5%(N=3).  ‘Help to deal with beliefs that nothing bad will happen again’ – 12.2%(N=5)/ 5%(N=2).  ‘Help to deal with other people’s expectations of me as a survivor’ – 15% (N=6)/ 5%(N=2). | Not reported. | ‘To feel I can manage my health together with my health team’ –10%(N=4)/2.4%(N=1). | ‘My doctors to talk to each other to coordinate my care’ – (10%(N=4)/9.8% (N=4).  ‘The very best medical care’ – 5.1%(N=2)/7.5%(N=3).  ‘My complaints regarding my care to be properly addresses’ – 7.3%(N=3)/4.9% (N=2).  ‘Understandable information’ – 2.5% (N=1)/4.9% (N=2).  ‘Local health services available when I require them’ – 5% (N=2)/ 2.5% (N=1).  ‘Up to date information’ 2.5% (N=1)/2.4% (N=1). ‘Information for family/others’ – 0% (N=0)/ 2.4% (N=1). | None | Not reported. | ‘Help with problems with my sex life’ – (10% (N=4)/(10.3% N=4).  ‘Help to adjust to change to the way I feel about my body’ – 7.5%(N=3)/ 7.5% (N=3). | Not reported. | ‘Help to know how to support my partner/family’ – 7.3%(N=3)/ 7.3%(N=3). | Unmet supportive care needs at T0/T2:  ‘To talk to other testicular survivors like me’ – 7.5%(N=3)/ 10% (N=4).  ‘Help to know how to deal with the impact of cancer on my relationships’ – 7.3% (N=3)/7.3%(N=3).  ‘Help to handle the topic of cancer in social/work situations’ – 4.9%(N=2)/2.5% (N=1).  ‘Help to make new relationships’ – 2.4% (N=1)/2.5% (N=1). | |
| Bender et al., 2012  62.5% of participants had one or more unmet needs  Younger age, seminoma and treatment with chemotherapy, radiotherapy, retroperitoneal lymph node dissection are associated with more overall needs (and met needs)  Unemployed patients were more likely to have unmet needs when compared to employed patients | Reports patients needing help with hair loss. Number unspecified. | ‘reduce stress’ – 27% (N=53).  ‘deal with my own of other’ expectations of me as a cancer survivor’ – 25.8% (N=51).  ‘manage my concerns about the cancer coming back’ – 25.3% (N=50).  ‘adjust to the changes in quality of life as a result of cancer’ – 19% (N=38).  ‘help to move on with my life’ – 19% (N=38) | Not reported. | Not reported. | ‘community support services’ –20.8% (N=41).  ‘access complementary or alternative therapy services’ – 21.6% (N=43).  Help with decision to get a prothesis. Numbers not provided.  Information for their family to know what to expect post after treatment. Numbers not provided. | Not reported. | Not reported. | ‘adjust to the changed I feel about my body’ – 27.6% (N=55). | ‘Find out about financial support of governmental benefits to which I am entitled’ – 28.1% (N=56). | Not reported. | ‘handle the topic of cancer in social and/or work situations’ – 20.7% (N=41).  ‘talk to others who have experienced cancer’ – 20.4% (N=41). | |
| Brand et al., 2015. | Concern about having one testicle – 10% (N=2).  Concern over physical appearance – 10% (N=2). | Not reported. | Not reported. | Not reported. | Information on sexual issues was provided to 78% (N=15) of TCS at diagnosis. Information had a 78% satisfaction rate.  Information on sexual issues was wanted by 58% (N=11) of participants at diagnosis and 40% (N=8) post diagnosis. | Not reported. | Not reported. | Concern about sex life – 25% (N=5).  Concerns about finding a partner – 10% (N=2).  Concerns about fertility – 30% (N=6).  Concern about testosterone level – 25% (N=5). | Not reported. | Concerns about the ability to have children (30%, N=6) | Not reported. | |
| **Author and Year** | **Physical Needs** | **Psychological/Emotional Needs** | **Cognitive Needs** | **Patient-Clinician Communication Needs** | **Health System/Information Needs** | **Spiritual Needs** | **Daily Living Needs** | **Interpersonal/intimacy Needs** | **Practical Needs** | **Family Related Needs** | **Social Needs** | |
| Bumbasirevic et al., 2013. | 18% (N=36) TCS had abnormally high levels of fatigue. | Emotional vitality was rated lowest in the EORTC and QLQ-30 (Mean =77.89).  Mild depression present in 10% (N=22) and moderate depression was present in 2% (N=4) of TCS.  Age is a risk factor for developing depression (OR03.2, 95 % CI 1.3–8.1, p00.012).  Depression is associated with fatigue (r=0.589, p=.001). | Not reported. | None reported. | Not reported. | Not reported. | Not reported. | 27.3% (N=55) of TCS reported worse sexual function compared to before treatment.  Self-reported impaired erectile function present in 20.8% (N=42) of TCS  Self-reported impaired ejaculation present in 25.7% (N=52) of TCS.  Loss of sexual desire reported by 17.3% (N=35) of TCS.  Ejaculatory dysfunction is associated with poor scores in bodily pain, general health, role-physical, physical functioning, composite scores and total scores in the Short Form 36. | Not reported. | Not reported. | Not reported. | |
| Darabos and Hoyt., 2017. | Not reported. | Clinical depression – 12.9% (N=22).  Anxiety disorder – 9.9% (N=17).  Future worry (b = -0.16, p < 0.05) and perceived stress (b = -0.36, p < 0.001) in testicular cancer survivors is linked to poor physical wellbeing [F(8,159) = 16.27, R2 = 0.44].  Physical wellbeing was associated with mental health history (anxiety (r = -0.30, p < 0.001), depression (r = -0.28, p < 0.001) | Not reported. | Not reported. | Not reported. | Not reported. | Not reported. | Not reported. | Not reported. | Not reported. | Not reported. | |
| **De Padova et al., 2011.** | 23% (N=10) of patients/caregivers reported ‘quite a lot/a lot’ of fatigue.  25% (N=11) of patients/caregivers reported ‘a lot/quite a lot’ of infertility. | 35% (N=15) of patients/caregivers reported psychological distress was ‘quite a lot/ a lot’ relevant.  42% (N=18) of patients/caregivers reported fear of recurrence was ‘high/very high’ in TCS. | Not reported. | 5% (N=2) of patients/caregivers reported experiencing ‘a lot’ of difficulties in relationships with their healthcare providers. | 90% of patients/caregivers reported websites about cancer and survivorship were ‘important/fundamental’. | Not reported. | Not reported. | 14% (N=6) of ‘patients/caregivers reported none/a little problems in sexual life’. | 19% (N=8) of patients/caregivers reported ‘quite a lot/a lot’ of difficulties in work and/or study. | 9% (N=4) of patients/carers reported experiencing ‘quite a lot/ a lot’ of problems in family relationships.  12% (N=5) of patients/carers reported experiencing strain in their relationship with their partner. | 14% (N=6) of patients/caregiver reported TCS social relationships were made ‘quite/very difficult’ by TC. | |
| Dimitropoulos et al., 2015. | Not reported. | Not reported. | Not reported. | Not reported. | Not reported. | Not reported. | Not reported. | PC-RPLND patients experienced decreased satisfaction from intercourse post-surgery (4.57 ± 0.80 vs 1.94 ± 0.67 (p=0.000)).  PC-RPLND patients experienced decreased sexual intercourse enjoyment post-surgery (4.57 ± 0.80 vs 2.06 ± 0.63 (p=0.000)).  PC-RPLND patients experienced decreased frequency of ejaculation post-surgery (4.96 ± 0.20 vs 1.21 ± 0.81 (p=0.000)).  PC-RPLND patients experienced decreased frequency of orgasm post-surgery (4.56 ± 0.19 vs 4.60 ± 0.69 (p=0.019)).  PC-RPLND patients experienced decreased satisfaction with overall sexual life post-surgery (4.34 ± 0.96 vs 1.69 ± 0.61 (p=0.000)).  PC-RPLND patients experienced decreased satisfaction with overall sexual relationship post-surgery (4.74 ± 0.44 vs 1.88 ± 0.77 (p=0.000)).  100% of PC-RPLND patients experienced total loss of antegrade ejaculation. | Not reported. | Not reported. | Not reported. | |
| **Author and Year** | **Physical Needs** | **Psychological/Emotional Needs** | **Cognitive Needs** | **Patient-Clinician Communication Needs** | **Health System/Information Needs** | **Spiritual Needs** | **Daily Living Needs** | **Interpersonal/intimacy Needs** | **Practical Needs** | **Family Related Needs** | **Social Needs** | |
| Kerns et al., 2020. | TCS who received cisplatin experienced:  Obesity – 69.1% (N=1254). Peripheral sensory neuropathy – 55.3% (N=1004). Patient -reported renal disease – 2.4% (N=44). eGFR-defined renal disease – 50.1% (N=489). Tinnitus – 39% (N=708).  Patient-reported hearing loss – 37.9% (N=688). Raynaud phenomenon – 32.5% (N=590). Autonomic neuropathy – 26.6% (N=483). Hypertension – 8.5% (N=154). Pain – 24.2% (N=440). Hypercholesterolemia – 6.8% (N=123). Hypertriglyceridemia – 0.4% (N=8). Thromboembolic event – 6.8% (N=124). Peripheral artery disease – 4% (N=72). Diabetes – 3% (N=54).  Thyroid disease – 2.6% (N=47). Coronary artery disease – 2.5% (N=45). Transient ischaemic attack – 0.6% (N=10). Stroke – 0.5% (N=9). Hypogonadism – 8.5% (N=154). | TCS who received cisplatin experienced:  Anxiety and/or depression – 5% (N=91). | Not reported. | Not reported. | Not reported. | Not reported. | Not reported. | TCS who received cisplatin experienced:  Erectile dysfunction – 26.9% (N=489). | Testicular cancer survivors were more likely to be unemployed when compared to population norms  Pain was more common in people on disability leave than those who were working full time | Not reported. | Not reported. | |
| Nord et al., 2015. | Not reported. | Not reported. | Not reported. | Not reported. | Not reported. | Not reported. | Not reported. | Not reported. | TCS take more sick leave than population comparators. Year after diagnosis - 64% (compared to 12%).  Risk of work loss persists to the third year of follow up  Patients who did not have more than 4 treatments did not experience work loss after 1 year. | Not reported. | Not reported. | |
| O’Carrigan et al., 2014. | 33% of TCS had hypogonadism. | Anxiety subscale Normal – 81% (N=44) Mild – 13% (N=7) Moderate – 6% (N=3) Severe – 0% (N=0)  Depression subscale Normal – 94% (51) Mild – 6% (N=3) Moderate – 0% (N=0) Severe – 0% (N=0) | Not reported. | Not reported. | Not reported. | Not reported. | Not reported. | Not reported. | Not reported. | Not reported. | Not reported. | |
| Oechsle et al., 2016. | Symptom frequency/rated distress:  Lack of energy (49%/21%), tiredness/drowsiness (42%/9%),pain (29%/8%),hair loss (13%/2%), sleep disturbances (36%/10%), itching (17%/4%), cough (17%/3%), sweats (29%/9%), shortness of breath (15%/3%), dizziness (15%/2%), skin changes (10%/3%), mucositis (9%/1%), numbness and tingling (29%/9%), feeling bloated (9%/3%), food taste (6%/3%).  Problems with urination (6%/2%). Constipation (5%/1%). Diarrhea (11%/2%).  Average number of physical symptoms – 4.5 (SD = 4.4; range, 1–28).  Unemployed patients (when compared to employed patients) (r = 0.28, p < 0.001), lower socioeconomic status (r = −0.20, p = 0.02) patients and older patients s (r = 0.19, p = 0.01) were more likely to experience more physical symptoms | Symptom frequency/rated distress:  Don’t look like self (4%/1%), irritability (47%), feeling worried (42%), sadness (27%), Nervousness (24%)  Average number of psychological symptoms - 1.4 (SD = 1.4) | Symptom frequency/rated distress:  Difficulty concentrating (32%/8%) | Not reported. | Not reported. | Not reported. | Not reported. | Reduced sexual interest (22%) | Not reported. | Not reported. | Not reported. | |
| Pallotti et al., 2019. | Hypogonadism was present in 4.1% of TCS and none of the controls. | Not reported. | Not reported. | Not reported. | Not reported. | Not reported. | Not reported. | Pre chemotherapy (post orchiectomy) - Erectile dysfunction present in 91/241 (37.8%) of TCS and 22/223 (9.9%) of controls.  Post chemotherapy erectile dysfunction returned to levels similar to the control group.  TCS experienced consistently worse sexual desire, intercourse satisfaction and general satisfaction than controls. | Not reported. | Not reported. | Not reported. | |
| Püse et al., 2012. | Some testicular cancer survivors experience chronic pain. Numbers not provided. Percentages not reported. Chronic pain effects sexual functioning. | Not reported. | Not reported. | Not reported. | Not reported. | Not reported. | Not reported. | TCS experienced:  Reduced sexual desire - 34.5%.  Reduced sexual activity – 41.6%. Erectile dysfunction – 31.5%.  Inability to maintain erection during intercourse – 24.4%. Ejaculatory disorders – 84.9%.  Reduced orgasm intensity - 32.4%. Reduced sexual satisfaction – 95.4%.  . | Not reported. | Not reported. | Not reported | |
| Shen et al., 2016. | Not reported. | 16.7% of TCS felt discouraged about their health problems ‘a good bit of the time’ or more frequently. N=values not provided.  Health distress scores were higher in TCS with education above secondary level (P = 0.031). 23.5% of TCS felt fearful about their future health ‘ a good bit of the time’ or more frequently. N=values not provided.  23.5% of TCS worry about their health ‘a good bit of the time’ or more frequently. N=values not provided.  29.4% of TCS are frustrated by their health problems ‘a good bit of the time’ or more frequently. N=values not provided.  68.7% (N=57/83) of TCS were satisfied with the emotional support from their providers of care. | Not reported. | 76% (N=57/75) of TCS felt their providers understood their expectations, beliefs and preferences.  74.1% (N=63/85) of TCS felt “known” by their care providers  91.9% (N=79/86) of TCS had confidence in the providers involved in their care.  80.8% N=59/73) of TCS felt prepared for discharge. | Young TCS (<40 years; P ¼ 0.013) and those who were not married or de facto (P < 0.045) have lower survivorship knowledge than those who are older and are married or de facto .  TCS younger than 40 years were less likely to report being given education, self-management tools and patient resources (P = 0.05) and had lower Information  Transfer and Management of Follow-up care scores (P = 0.05).  43.3% of TCS know about supports available to them. N=values not provided.  58.8% of TCS know how to ‘manage their health risks’. N=values not provided.  58.9% of TCS ‘know the chances or their cancer coming back’ and the ‘likelihood of them getting another type of cancer’. N=values not provided.  64.5% of TCS ‘know the health risks and long-term effects’ of their cancer treatments. N=values not provided.  74.1% of TCS know ‘what screening tests’ they need to undergo. N=values not provided.  76.6% of TCS ‘know the stage’ of their cancer. N=values not provided.  85.3% of TCS know what treatments they have undergone. N=values not provided.  87.8% of TCS’ know what doctor’ they need to see and the frequency they need to see them. N=values not provided.  90.8% (N=79/87) of people were ‘provided clear information’ on their diagnosis  87.5% (N=77/88) were ‘provided clear information’ on prognosis  61.8% (N=47/76) were ‘told about nonurgent symptoms that may occur and how to cope with them’  71.2% (N=52/73) were given information on ‘symptoms that require urgent medical attention and who to contact if they occur’  79.6% (N=39/49) were ‘given complete information; on their medications  94.1% (N=80/85) were ‘given information on follow up appointments’  81.1% (N=60/74) were ‘informed of ongoing treatment that may be necessary after discharged and whether they would have ongoing contact with providers of my care’  76.8% (N=63/82) had ‘a well developed and realistic follow up care plan prepared and explained’ to them.  29.3% (N=22/75) were ‘informed of self-management tools and education materials’ that could help them  39.2% (N=31/79) were ‘informed of patient resources/supports that may be available.’  31.8% (N=21/66) report their ‘informal caregivers were given information on resources/supports’ | Not reported. | Not reported. | Not reported. | Not reported. | Not reported. | Not reported. | |
| **Author and Year** | **Physical Needs** | **Psychological/Emotional Needs** | **Cognitive Needs** | **Patient-Clinician Communication Needs** | **Health System/ Information Needs** | **Spiritual Needs** | **Daily Living Needs** | **Interpersonal/intimacy Needs** | **Practical Needs** | **Family Related Needs** | **Social Needs** | |
| Skaali et al., 2011a. | Not reported. | Not reported. | N=42/122 TCP had a cognitive decline of more than >10% from baseline. Associated with increased hearing loss/tinnitus (p=0.03). Not related to any other variables. | Not reported. | Not reported. | Not reported. | Not reported. | Not reported. | Not reported. | Not reported. | Not reported. | |
| Skaali et al., 2011b. | Neurotoxic symptoms (SCIN): Peripheral neuropathy, baseline/follow-up: 4% (N=5)/15% (N=18) Raynaud-like symptoms, baseline/follow-up: 9% (N=11)/25%(N=31) Tinnitus or hearing loss, baseline/follow-up: 9% (N=11)/25%(N=31) | Not reported. | Testicular cancer treatment resulted in a neuropsychological decline in 34% (N=42) patients. This was not found to have statistically significant related to increase in self-reported cognitive problems (*P=.*82)  20% (N=25) of TCP had more self reported cognitive functions at follow up when compared to baseline. They were also more likely to have received chemotherapy (96% vs 69%), experience Raynaud-like symptoms (42% vs 16%), have a lower education level (68% vs 44%), a history of psychological problems (48% vs 19%), increased fatigue score (50% vs 22%).  More TCPs who received a single (29%) or multiple chemotherapy sessions (25%) reported increased cognitive problems than TCPs who did not receive chemotherapy.  Increased self-reported cognitive problems were positively associated with psychological distress | Not reported. | Not reported. | Not reported. | Not reported. | Not reported. | Not reported. | Not reported. | Not reported. | |
| Skaali et al., 2011c. | Not reported. | 24% of newly diagnosed TCS had cancer related distress (95%CI 17%-31%). | Time used on Color-Word Interference Test (CW) 1 (used to measure psychomotor speed) and 3 (used to measure executive function) was associated with Impact of Events Scale (IES) scores (used to measure trauma) (adjusted p=0.04, adjusted p=0.03). | Not reported. | Not reported. | Not reported. | Not reported. | Not reported. | Not reported. | Not reported. | Not reported. | |
| Smith et al., 2013.  66% of TCS reported one or more unmet needs  Mean number of unmet needs - 4.73 (SD = 7.0, Range = 0–34)  Unmet needs per domain: Information–0.32 (SD = 0.76), Relationships–0.50 (SD = 0.90), and QoL–0.26 (SD = 0.61)  Chronic illness (b = 0.189, p = 0.01, sr2 = 0.03) and young age (b = 0.188, p = 0.04, sr2 = 0.02) was associated with increases number of unmet needs (b = 0.189, p = 0.01, sr2 = 0.03)  Radiotherapy was positively related to need strength (b = 0.161, p = 0.04, r2 = 0.03)  Chemotherapy was negatively associated with need strength (b = 0.196, p = .01, r2 = .04). | Not reported. | TCS reported needing [Mean (CI) strength rating]:  ‘help to reduce stress in my life’ – 30% (N=72/239) [1.89 (1.71,2.07)]  ‘help to manage concerns about my cancer coming back’ – 22% (N=52/239) [1.79 (1.59,2.21)]  ‘help to cope with others not acknowledging the impact that cancer has had on my life’ – 18% (N=42/240) [1.71 (1.45,197)]  ‘help to deal with my own and/or others expectations of me as a “cancer survivor”’ – 17% (N=41/240) [1.63 (1.39,1.88)]  ‘emotional support to be provided to me’ – 17% (40/240) [1.80 (1.55,2.05)]  ‘help to make my life count’ – 13% (N=32/240) [2.03 (1.76,2.30)] | Not reported. | TCS reported needing [Mean (CI) strength rating]:  ‘to know that all my doctors talk to each other to coordinate my care’ – 14% (N=33/238)  ‘to feel like I am managing my health together with the medical team’ – 8% (N=19/238) [2.12 (1.76,2.48)] | TCS reported needing [Mean (CI) strength rating]:  ‘the very best medical care’ – 7% (N=17/239) [2.47 (2.10,2.84)]  ‘any complaints regarding my care to be properly addressed’ – 9% (N=21/238) [2.29 (1.96,2.61)}  Mean number of unmet information needs information–0.32 (SD = 0.76)  ‘local health care services that are available when I require them’ – 11% (N= 26/239) [2.08 (1.76,2.40)] | Not reported. | Not reported. | TCS reported needing [Mean (CI) strength rating]:  ‘help to address problems with my/our sex life’ – 23% (N=56/240) [1.76 (1.56,1.97)]  ‘help with having a family because of fertility problems’ – 11% (N=26/236) [2.35 (2.05,2.65)] | TCS reported needing [Mean (CI) strength rating]:  ‘help to find out about financial support or government benefits to which I am entitled’- 22% (N=52/239) [1.98 (1.75,2.21)]  ‘getting life and/or travel insurance because of my cancer’ – 20% (N=42/240) [2.08, (1.86,2.31)]  ‘more  accessible hospital parking’ – 17% (41/239) [2.05 (1.78,2.32)] | Not reported. | TCS reported needing [Mean (CI) strength rating]:  ‘help to talk to ithers who have experienced cancer’ – 17% (N=41/240) [1.68 (1.43,1.93)]  Mean number of unmet supportive care needs related to relationships 0.50 (SD = 0.90), | |
| Smith et al., 2016. | TCS have lower mean physical functioning than the age and gender adjusted Australian general population mean (52.82/53.32).  TCS have lower mean physical role functioning than the age and gender adjusted general population (50.44/52.02).  TCS have higher mean bodily pain than the age and gender adjusted general population (52.86/51.31).  TCS have lower mean general health than the age and gender adjusted population (48.48/ 51.44).  TCS have lower mean vitality than age and gender adjusted population (48.59/51.93).  Low health related quality of life is positively rated to job problems (β=−0.38, p<0.001)  31% of TCS reported high levels (“very much” or “quite a bit”) of concern about their fertility. (N=values not provided). | TCS had higher mean levels of depression (48.64, SD=10.06)  vs. 51.07, SD=12.78; t(243)=2.97, p=0.003) and anxiety (48.27, SD=10.19 vs. 50.73, SD=14.07; t(243)=2.73, p=0.007) than the age adjusted population.  TCS had lower mean emotional role functioning than age and gender adjusted populations (44.97/51.10).  TCS had lower mean mental health than the age and gender adjusted populations (44.79/50.81).  TCS had lower mean mental component summary scores than the age and gender adjusted populations (43.59/50.44).  30% of TCS report high levels (“very much” or :quite a bit”) of fear of recurrence. (N=values not provided).  29% of TCS reported high levels (“very much” or “quite a bit”) of uncertainty about the future. (N=values not provided).  26% of TCS reported high levels (“very much” or “quite a bit” of concern around the disruption testicular cancer had caused in their lives. (N=values not provided).  15% of TCS reported high levels (“very much: or “quite a bit”) of concern about their masculinity because of their diagnosis and treatment. (N=values not provided).  Total support was negatively associated with stress (β= −0.25, p=0.001) and depression (β=−0.18, p=0.003)  Helpless coping style as positively associated with depression (β=0.46, p<0.001), stress (β=0.23, p=0.007), and anxiety (β= 0.20, p=0.003).  “Unmet needs uniquely explained 2 % of the variance in both depression (β=0.19, p=0.002) and anxiety (β= 0.20, p=0.003)”.  Low mental health quality of life was associated with reduced sexual activity (β=0.22, <0.001,.helpless/hopeless coping style a helpless/hopeless coping style (β=−0.31, p<0.001) and low social support (β=0.21, p=0.001) | Not reported. | Not reported. | Not reported. | Not reported. | Not reported. | 27% of TCS reported being “not at all” or “a little bit” interested in sex. (N=values not provided).  41% of TCS reported being “not at all” or “a little bit” sexually active  27% of TCS reported being able to talk to their partner or the person closest to them about sex “a little bit” or “not at all”. (N=values not provided).  22% of TCS who received a prosthesis were “a little bit” or “not at all” satisfied with their outcome. (N=values not provided). | Not reported. | Not reported. | TCS have lower mean social functioning than the age and gender adjusted population (48.09/51.12). | |
| Soleimani et al., 2021. | Physical concerns of AYA and non-AYA patients were not different in a statistically significant way. Shared concerns included sleep 24.7% vs. 25.4%, p = 0.879), weight (17.2% vs. 13.1%, p = 0.320)” and concentration/memory (18.9 vs. 11.5%, p = 0.072). | 35.2% (N=123) of TCS had self-reported symptoms of anxiety  AYA had higher rates of self-reported anxiety symptoms (39.2% N=89) than non-adolescents and young adults (27.9% N=34)  AYA reported more frustration/anger than non-adolescents and young adults (26% vs. 16.4%, p = 0.041) | Not reported. | Not reported. | 37.9% of AYA and 36.1% non-AYA, p = 0.74) were concerned about their understanding of their illness and the treatments they had undergone. | The least concerning areas for both AYA and non-AYA were faith (11.2% vs. 0.7% p < 0.001) and the meaning of  life (12.4% vs. 2.2% p = 0.002) | Not reported. | Not reported. | More AYA had concerns about work and/or school than non-AYA (38.3% vs. 18.9%, p < 0.001)  More AYA had concerns about finances than non-AYA (34.9% vs. 18.9%, p = 0.002) | More AYA reported being worried about their family and friends than AYA (21% vs 19.7%) (p= value not reported) | Not reported. | |
| Stouten-Kemperman et al., 2015. | White matter hyperintensities were higher in TCS treated with chemotherapy [χ^2^ (2, N = 45) 5 5.29, P = 0.07].  Radial kurtosis was higher in TCS treated with chemotherapy (F_143_= 4.36, p= 0.043). | TCS treated with chemotherapy had more body change concerns han those treated with surgery alone (p= 0.003). | TCS treated with chemotherapy had more self reported cognitive problems than those treated with surgery alone (35.7 vs. 4.3%, v2  (2, N = 51) = 7.34, P = 0.007)). | Not reported. | Not reported. | Not reported. | Not reported. | Not reported. | 14.8% (N= 4) of TCS treated with chemotherapy reported that workplaces changes were necessary to facilitate their return to work. Not statistically significant.  11% (N=3)of TCS treated with chemotherapy got a new job. Not statistically significant. | Not reported. | Not reported. | |
| Tasdemir et al., 2012. | Not reported. | TCS who had undergone chemotherapy had higher levels of anxiety than the general population (mean ± standard deviation) 45.0 ± 12.7 vs 10.1 ± 6.8 (P < 0.05). (N= values not reported). | Not reported. | Not reported. | Not reported. | Not reported. | Not reported. | TCS who received chemotherapy were more likely to experience erectile dysfunction than healthy controls P < 0.05) | Not reported. | Not reported. | Not reported. | |
| Vehling et al., 2021**.**  33% of TCS report one or more negative changes [mean = 1.1 (SD = 2.5)] | Mean physical symptom count (SD) – 4.5 (4.3) | TCS reported negative life changes including:  Negative life changes were reported by 33% of TCS. On average 1.1 changes was reported (SD = 2.5).  Depression (mean): Low to mild – 92.1% (N=151). Moderate to high – 7.9% (N=13).  Anxiety (mean): Low to mild -93.9% (N=154). Moderate to high – 6.1% (N=10). | Not reported. | Not reported. | Not reported. | Not reported. | Not reported. | Not reported. | Not reported. | Not reported. | Not reported. | |
| Vehling et al., 2016. | 49% of TCS report lack of energy. (N= value not reported).  42% of TCS report feeling drowsy. (N= value not reported).  36% of TCS report sleeping problems. (N= value not reported).  Increased levels of physical symptoms were associated with increased anxiety and depression (anxiety: b=0.55, 95% CI=0.41 to 0.69, P≤0.001, depression: b=0.62,  95% CI=0.49 to 0.75, P≤0.001). | Moderate anxiety was present in 6% (N=10) of TCS.  Moderate depression occurred in 8% (N=13) of TCS.  Anxiety and depression were more likely to occur if a survivor had children [anxiety:  b=0.43, 95% confidence interval (CI)=0.15 to 0.71, P=0.003, depression: b=0.37, 95% CI=0.11 to 0.62, P=0.006].  Younger age and diagnosis is associated with increased anxiety (b=−0.21,  95% CI=−0.37 to −0.06, P=0.01).  Increased time since diagnosis was associated with lower anxiety (b=−0.15,  95% CI=−0.29 to −0.01, P=0.04).  Negative life changes are associated with anxiety (β = 0.23, 95% CI 0.11 to 0.36), and depression (β = 0.15, 95% CI −0.03 to 0.27) | Not reported. | Not reported. | Not reported. | Not reported. | Not reported. | Not reported. | Not reported. | Not reported. | Not reported. | |
| Wang and Hoyt, 2020. | Not reported. | Cancer related masculinity threat score in TCS (M = 2.43, SD = .64)  Depression score in TCS (M = 12.99, SD = 12.08)  Negative affect score in TCS (M = 1.80, SD = .74)  Cancer related masculinity threat was associated with negative affect (r = .50, p < .001) and depression (r= .50, p < .001). | Not reported. | Not reported. | Not reported. | Not reported. | Not reported. | Not reported. | Not reported. | Not reported. | Not reported. | |
| Wefel et al., 2014. | Not reported. | Not reported. | TCS who received chemotherapy experienced cognitive decline (on more than two tests) post treatment (low exposure – 17% vs high exposure – 29%). The surveillance group had no decline. Not statistically significant when compared to surveillance group (p=0.280, p= 0.08).  Overall decline (on more than two tests) from post treatment to 12 month follow up occurred in 52% of TCS who had low exposure to chemotherapy and 67% of TCS who had high exposure to chemotherapy. Statistically significant when compared to surveillance group (p=0.006, p=0.001)  Young age was associated with cognitive decline. | Not reported. | Not reported. | Not reported. | Not reported. | Not reported. | Not reported. | Not reported. | Not reported. | |
| Wefel et al., 2011. | Not reported. | 10% (N=7/69) of TCP had depression and 7% (N=5/69) TCPs had anxiety. | 46% (n=32/69, p=0.0001) were considered cognitively impaired. | Not reported. | Not reported. | Not reported. | Not reported. | Not reported. | Not reported. | Not reported. | Not reported. | |
| Wortel et al., 2015. | 48% of TCSs reported concerns about fertility. 23% had concerns that were moderate – severe. More common in young patients (Spearman’s r=-0.555, p=<0.001). | 51% of TCS reported minor changes in body image post orchiectomy. An additional 10% reported moderate – severe changes. No correlation with age (p=.233). Not present in prothesis patients.  19% of TCS reported concerns about undressing around other men after orchiectomy. More common in young patients (Spearman’s r=-0.194, p=0.014). Not present in prothesis patients. | Not reported. | Not reported. | 44% of TCS had not received information on prosthesis. | Not reported. | Not reported. | When compared with baseline surveys, 6 months surveys indicate reduced erectile rigidity in TCS (p=0.016, 33% vs 25%).  Body image changes were correlated with lower sexual functioning (erectile rigidity p=0.032, sexual pleasure p=0.021, sexual interest p=0.043, erectile function p=0.002)  23% of TCS had a reduction of sexual pleasure, sexual interest and activity six months post radiotherapy (p= 0.01).  45% of TCS reported negative effects on sex life. N=14 had moderate tp severe effects.  Poor body image was had adverse effects on sex life (Spearman’s r=0.267, p=0.003).  Erectile rigidity, sexual interest, sexual satisfaction, erectile dysfunction, sexual pleasure and sexual activity were associated with worse sexual life (p=<0.01).  13% of TCS reported experiencing sexual difficulties with their partner due to have one testicle. More common in young patients (Spearman’s r=-0.225, p=0.004). Not present in prothesis patients. | Not reported. | Not reported. | Not reported. | |

Testicular cancer survivors (TCS), testicular cancer (TC); testicular cancer patients (TCP); adolescents and young people (AYA).
