# Supplementary material for: Identifying the unmet supportive care needs of individuals affected by testicular cancer: a systematic review
Source: J Cancer Surviv. 2022 Jul 4;18(2):263–87. doi: 10.1007/s11764-022-01219-7 (PMC10960773; doi:10.1007/s11764-022-01219-7)
Supplement: Supplementary file 4 — Supplementary file4 (DOCX 40 KB) [file 11764_2022_1219_MOESM4_ESM.docx]

**Table 9: Study Findings and Illustrations**

| **Authors: Carpentier et al., 2011** |  |  | | |  |  |  |
| --- | --- | --- | --- | --- | --- | --- | --- |
| **Findings** | **Illustrations (Page number)** | | **Evidence** | | | | **Finding number** |
|  |  |  | **Unequivocal** | **Credible** | | **Unsupported** |  |
| **Adolescents and young adults are embarrassed by their symptoms of testicular cancer and delay seeking care as a result.** | When I went to the emergency room, the check-in desk at the emergency room, there was two young girls working there. I’m only 25 years old and they kept asking me what the problem was and I kind of pointed down there… I was embarrassed to say that I have a big knot down there’ (Participant 19)” (page 741). | |  | **Yes** | |  | **1** |
| **Masculinity prevented testicular cancer survivors from seeking immediate care** | “‘I happened to just check one day and I was like ‘Oh!’ and a month later I went to the doctor it’s just the stubbornness of a guy, okay, it’s not a big deal’ (Participant 2) (page 741)  “‘Most guys have that ego where, ah, it’s probably nothing. I had that same ego at the time, I’ve never had the flu or anything, so I’m like there’s no way I’m sick. Yeah, I’d definitely say it’s the masculinity that gets in the way’ (Participant 17).” (page 741) | | **Yes** |  | |  | **2** |
| **Adolescents and young adults who had testicular cancer feel different from their peers as a result of their cancer.** | “‘You consider life a little bit differently and your realities would be for a shorter time period instead of longer so, yeah, there are some changes, especially on my values’ (Participant 4)” (page 741).  “‘I look at things differently than a lot of people though, so I’m kind of a little bit on the abnormal area, I suppose’ (Participant 1)” (page 741).  “‘They know that I had cancer, but to them, if it didn’t kill you then you’re fine… they don’t know how I feel, they don’t know how I think, they don’t know what it feels like but they’ll tell you ‘well, you ought to do this or I would do this.’ They don’t know, they don’t have a clue’ (Participant 19).” (page 741)  “‘When I got married I guess because of being a cancer patient I felt like the marriage was, I took it a little more seriously than I might have before’ (Participant 1)” (page 741)  “‘It was better for our relationship, it made us realize that we wanted to be together, and so settled me down from, got me more focused on maturing and growing up, stuff like that. So, if anything, it was better for our relationship.’”  “‘They said one of the things that can happen is infertility, and I thought with all the health problems that I had, I do not want to pass it onto somebody else, no, no, no, definitely not. My thought is if they ever wanted kids, I would probably adopt, as opposed to making my own… I guess it’s just something I will deal with when the time comes’ (Participant 13).” (page 741-742) | |  | **Yes** | |  | **3** |
| **Adolescents and young adults felt that being different made them “damaged goods”.** | “‘Losing my hair was probably more devastating than losing my testicle I think. Because I went from liking my hair to having none in about three days. And that was a big adjustment. Even though a lot of guys you see on the street shave their head, and have short hair when it first happened midway through the chemotherapy… I hadn’t shaved in three or four days or whatever, it was just falling off. That was probably as devastating as anything, for me. It was just like, ‘wow’ (Participant 1)” (page 742).  “‘Because the scar, especially from the radical lymph node dissection, is quite obvious. It’s like, a twelve inch scar is hard to hide. That’s probably the biggest problem. Is that even if I did not want to tell anyone, there’s no point in lying’ (Participant 9)  ‘It just feels kind of like you’re incomplete. Just as a person you feel like you’re missing something you’re supposed to have. I guess it’s just the fact that it doesn’t have any real effect but there’s still something missing. So it’s just that weird dichotomy’ (Participant 9)” (page 742).  “‘It’s just the part about being a man and the man having that ability to, and also losing part of that is like losing a part of yourself’ (Participant 2) (page 742)  ‘There was a time in the first intimate moments and you’ve had your testicle removed, you’re a little unsure of yourself’ (Participant 1)” (page 741).  “‘It’s something I’ve always had so losing any kind of body part, organ or anything just, you know you’ve only got one left. So, if it gets compromised and it has to be taken out at least I guess it’s not a lung or’ (Participant 6)” (page 741). | |  | **Yes** | |  | **4** |
| **Adolescents and young adults find disclosing their history of cancer challenging** | “‘I do have a few hang-ups about it, just saying that I have cancer to new people. Pretty much towards potential romantic partners. Because in class I don’t have a problem if we’re discussing something like that, it doesn’t really bother me. But, of course, usually in class I don’t specify what type just because in my mind it takes it to a much more intimate conversation than if it was skin cancer’ (Participant 9)” (page 742).  “‘I always wonder if it’s a first thing or, I don’t know. I guess it kind of depends on who the person is at this point, because I don’t really since I haven’t told anyone yet, I don’t really have any baseline to say, ‘well that was a really bad idea to say on the first date.’ Or, ‘wow, they got really mad because I didn’t tell them until two months into the relationship’(Participant 9)” (page 742).  “‘I kind of don’t want to drag anybody into all of this stuff right now. It’s a lot of stuff to swallow, going through all of it. I guess my point of view is that I always just thought who would want to get involved right now?’ (Participant 5)” (page 742)  “‘I had friends joking with me and stuff about it. It was all in good fun I guess but yeah sometimes it bothered me. I’m thinking, I’m fine now. You really can’t tell it’s not there’ (Participant 6).” (page 742) | | **Yes** |  | |  | **5** |

| **Authors: Martin et. al., 2013** |  |  | | |  |  |  |
| --- | --- | --- | --- | --- | --- | --- | --- |
| **Findings** | **Illustrations (Page number)** | | **Evidence** | | | | **Finding number** |
|  |  |  | **Unequivocal** | **Credible** | | **Unsupported** |  |
| **Patients need information needs regarding their health and healthcare are not adequately met.** | ““In that workshop . . . I found out more than I have done in the last probably six years of going through this.”” (Page E19)  ““No one ever told me about self-examination.”” (page E19)  ““My doctors have said, you know, live healthy and that but, you know, that workshop went a bit more into how to live healthy.”” (page E20)  ““I just wish I had it when I first started going through it and I never did.”” (page E21) | |  | **Yes** | |  | **1** |
| **An educational workshop met social needs of the testicular cancer survivors** | ““raises the whole agenda of how do you help patients to feel . . . that other people have been or are in the same situation.”” (page E20)  ““useful parts were actually having the opportunity to listen to other people, and it’s good to get the group talking about it.”” (page E20)  ““To see how the others responded to it highlighted for me just how important it was . . . because some of them had had quite traumatic experiences and, therefore, to be able to verbalize it and find there are others who could empathize with that, and also to be give some direction to say, “Look, you can begin to manage that.” I thought it was good.”” (page E20)  ““to be aware particularly of those that were on the same workshop whose conditions had been dramatically worse than mine and how they’ve, you know, responded to that actually put it back into perspective again.”” (page E20)  ““it’s probably the one time where we will open up. . . . So the opportunity to discuss things is what’s needed.” The group was relaxed, which was important because “we can have a laugh and talk about it, it just made it more of a social event as well.”” (page E20) | |  | **Yes** | |  | **2** |

| **Authors: Matheson et. al., 2016** |  |  | | |  |  |  |
| --- | --- | --- | --- | --- | --- | --- | --- |
| **Findings** | **Illustrations (Page number)** | | **Evidence** | | | | **Finding number** |
|  |  |  | **Unequivocal** | **Credible** | | **Unsupported** |  |
| **Testicular cancer survivors expressed that it would have been beneficial or was beneficial to speak to someone.** | “(‘that would have been nice to have had a mentor’ P18, T2, 44 years, single, Surgery + C + R)” (page 200)  “‘you can’t bottle it all up’ P23, T1, 30 years, in a relationship” (page 199)  “‘I just think it just helped just reassure me, like I wasn’t a nutter, or some weirdo, and you’re not the only person, you won’t ever be the only person who’s gone through it’ (P20, T1, 22 yrs, Single, Surgery+C)” (page 199) | |  | **Yes** | |  | **1** |
| **Some testicular cancer survivors required more information around which members of the healthcare team to contact when they required help** | “You just want someone to go, if you’ve got a problem go to your GP, or if not you phone this person, any questions phone this person, that would be the only thing, [health professionals’] just kind of go ‘well you’re fine you’re fine’ (P10, T1, 41 yrs, Married, Surgery)” (page 200) | | **Yes** |  | |  | **2** |
| **Some testicular cancer survivors report struggling with mental health when returning to work** | “‘some days you feel absolutely fine and silly little things remind you that you actually had cancer…. for a while I felt low and yeah I admit to my wife I’d thought at some stages I was maybe suffering from depression….I felt not lonely, sort of very pressurised and was very snappy, and then all of a sudden out the blue you have a good week, two weeks and things are fine, but things easily build, got on top of me very quickly, so I’d come back down again, no I never went to the doctors or anything, from time to time I still do get these times, but I’m assume that’s life of living with cancer, and a young family and pressures of life’ (P1, T2, 36 yrs, Married, Surgery+R*)” (page 201) | |  | **Yes** | |  | **3** |
| **When testicular cancer survivors experienced changes to their physical body due to their treatment, it affected their body image** | “(‘you kind of lose a little bit of your identity’ P2, T2, 24 years, single, Surgery + C*)” (page 201) | |  | **Yes** | |  | **4** |
| **Some testicular cancer survivors struggled with their sense of identity** | “‘I don’t at times feel good about myself, and then that worries me that I’m perhaps not as good as I should be for my wife and my child and everything else, and it does bother me.. but there doesn’t seem to be an avenue you can go, to sort it’ (P10, T2, 41 yrs, Married, Surgery)” (page 201) | | **Yes** |  | |  | **5** |
| **Some testicular cancer patients expressed sadness** | “(‘I wouldn’t say I’m back to total normal, no, not quite, I still don’t feel right’ P10, T2, 41 years, married, Surgery)” (page 202) | |  | **Yes** | |  | **6** |
| **Some testicular cancer patients expressed that they felt their health was vulnerable** | “(‘wait for the next thing to give up’ P10, T2, 41 years, married, Surgery)” (page 202) | |  | **Yes** | |  | **7** |
| **Some testicular cancer patients felt resentment as a result of infertility brought on by treatment** | “‘Just the resentment, you can’t be as you were… that one’s a bit more of a difficult one to get over, lots of our friends are obviously having babies…which is something that we feel that we’re more than prepared to do… so I know that I’ll never be able to look back on the cancer and think oh well that was a bit tough but I’m so glad it happened, there’s always going be a bit of hatred, bit of resentment there’ (P14, T2, 31 yrs, Married, Surgey+C*)” (page 202) | | **Yes** |  | |  | **8** |

| **Authors: Saab et al., 2016** |  |  | | |  |  |  |
| --- | --- | --- | --- | --- | --- | --- | --- |
| **Findings** | **Illustrations (Page number)** | | **Evidence** | | | | **Finding number** |
|  |  |  | **Unequivocal** | **Credible** | | **Unsupported** |  |
| **Testicular cancer survivors did not want to disclose their diagnosis.** | ““My sister doesn’t know that I got sick with cancer...she still doesn’t know. I took chemotherapy and lost my hair...I didn’t want anyone to see me and go tell my sister.”” (page 206) ““My biggest worry was that I don’t want my parents to know about the subject...I told them that I have to undergo a surgery because I have a kidney stone...I took chemotherapy and told them that I am losing my hair while showering....”” (page 206) ““I didn’t want to tell anyone...especially here in our village, if they knew that I was sick, the news would spread in the whole village. Thank God, nobody knows.”” (page 206) | | **Yes** |  | |  | **1** |
| **Testicular cancer and it’s treatments resulted in significant changes to the survivors lifestyle and outlook on life.** | ““After chemo, there was a time I used to play football but then I felt that my knees cannot hold me anymore so I stopped.”” (page 206)  ““Up until now, from the time I do a CT scan...and the alpha feto [alpha-fetoprotein], I feel that I am in a different world.”” (page 206) ““The doctor told me to do them [follow-up tests] every six months, but I am doing them every three months for reassurance.”” (page 206)  ““My life changed, it became healthy. I stopped smoking and stopped alcohol...I followed a totally different diet.”” (page 206)  ““As long as I have a good health and as long as my outlook on life is right, I want to move forward and not take a step back...I forgot about the past...I have to evolve and grow; I can’t remain the same.”” (page 206) | | **Yes** |  | |  |  |
| **Testicular cancer and its treatments caused infertility and affected the survivors confidence.** | ““Chemotherapy took my fertility away...I can’t have kids anymore.”” (page 206)  ““An infertile man... the way people perceive him makes him want to beat himself... I suffered...a man is about sex and kids to a certain extent.”” (page 206) ““If I want to propose to a girl and she would know that I have only one testicle, she might reject me.”” (page 206)  ““My doctor told me to do a sperm count, I refused...the result would affect my psychological well-being....”” (page 206) | | **Yes** |  | |  |  |

| **Authors: Shen et al., 2016** |  |  | | |  |  |  |
| --- | --- | --- | --- | --- | --- | --- | --- |
| **Findings** | **Illustrations (Page number)** | | **Evidence** | | | | **Finding number** |
|  |  |  | **Unequivocal** | **Credible** | | **Unsupported** |  |
| **Testicular cancer survivors felt they were not prepared for what to expect after treatment** | ““I don’t know what to look for, I don’t know what to expect.” (page e16)  ““I don’t know what the symptoms would be if the cancer came back… I wasn't given any of that detail. I wasn't told how I should change my lifestyle, if I should change my lifestyle. They just said, keep on living the way you do.”” (page e16)  ““Based on my personal experience, I don’t think we’re exactly prepared for after treatment, because it's kind of like, ‘whatever you have during treatment, that's the symptoms you're going to have after treatment. And we don't know how long it's going to take to come out- to go away, so deal with it until then.”” (page e16) | | **Yes** |  | |  | **1** |
| **Testicular cancer survivors had challenges in accessing reliable information and resources** | ““I saw a lot of things that really freaked me out on the internet that I probably shouldn't have looked at, and I wish that there was a specific guide or a specific like chapter that they refer me to... Don't start looking at all this other stuff… it spirals out of control. I was panicking…”” (page e16)  ““There was no discussion that I remember that was any, you know, ‘if you're feeling like this, then come and talk to us,' or, you know, ‘there's counseling available,' or anything like that. I don't recall anything like that for the psychological side of any concerns, really.” “The attitude seemed to be, if something bothers you, tell us and we'll deal with it. We're not going to tell you in advance what any of those things might be.”” (page e16)  “”There was no discussion that I remember that was any, you know, ‘if you're feeling like this, then come and talk to us,' or, you know, ‘there's counseling available,' or anything like that. I don't recall anything like that for the psychological side of any concerns, really.”” (page 16)  ““The onus always seemed to be on me to get in touch” “I think it would be great to have a document that contains all that information put together in a personalized way, just so the patient is aware of everything.”” (page e16)  ““I think it would be great to have a document that contains all that information put together in a personalized way, just so the patient is aware of everything.”” (page 16) | | **Yes** |  | |  | **2** |
| **Testicular cancer survivors experienced emotional difficulties** | ““…the bounce back from this was something that I couldn't cope with emotionally because I've never really dealt with a lot of emotions…I'm a guy…you need to be strong and that's what I was taught and you just deal with it and suck it up…”” (page e16)  ““By I think about three or four months [after going back to work] I just (clap!) I hit a wall and then all these emotional things came and I'm like whoa!” “I was fighting so long and then that died and then… a lot of emotional stuff came in.”” (page e16) | | **Yes** |  | |  | **3** |
| **Testicular cancer survivors did not feel reassured by healthcare providers** | ““I felt sort of brushed aside. I had questions that weren't answered, like that were almost basically ignored.” “It's great to find out everything's all right, but I think you need a little bit more.”” (page e16) | |  | **Yes** | |  | **4** |
| **Testicular cancer survivors wanted clearer communication about who to contact when they had concerns about their health** | ““So if I have a symptom that… you have a list of these possible symptoms… and I know to contact a particular person at the… hospital… who can tell me ‘you know what, you should go see your family doctor or, no, come see us.'”” (page e16) | | **Yes** |  | |  | **5** |

| **Authors: Wibe et. al., 2012** |  |  | | |  |  |  |
| --- | --- | --- | --- | --- | --- | --- | --- |
| **Findings** | **Illustrations (Page number)** | | **Evidence** | | | | **Finding number** |
|  |  |  | **Unequivocal** | **Credible** | | **Unsupported** |  |
| **Patients had unmet information needs** | ““I was at a 4 weeks’ check-up with you last week. Took some blood tests then, which showed some values that were too high. I was originally supposed to come back to you after 6 weeks, but now I’ve got a new appointment already after 2 weeks. How should I interpret this—a bigger chance that there is something in my body? Many people say that these values on blood tests go up and down. So I am very unsure what to think. Can I say that I don’t have cancer any more now, until something more is discovered?” (message)” (page 3)  ““... It (the information on the Internet) was a little contradictory ... [...] It said that there are two types (of testicular cancer): there is non-seminoma and there is another type. And one of them is bad and the other is not that bad. But as long as you don’t know what you have, it is really better not to read it, because ... I at least, became more worried. You start to think about the worst case, you know ...” (interview)” (page 4)  ““It’s usually at the start of an illness that you have questions, and perhaps some extra need for support [...] ... Like after my first surgery at the local hospital and before I was admitted to the regional hospital—who could I talk to in the meantime?”” (page 5)  ““... Yes, there was much that was still not clear, but on the other hand it would have been better to hear that “This is not clear yet, so you will have to wait until we have an answer to this and that before we can tell you”, rather than just: “We cannot answer you”. (interview)” (page 5) | |  | **Yes** | |  | **1** |
| **Patients felt uncomfortable discussing sexual function** | ““Sexual questions for example, which might have come up during the doctors’ rounds ... This might be easier to ask about in an e-mail to a person that you don’t know than when the doctor asks: “What about your ... (sexual function)?” Then you answer: “Oh, that’s OK” or “That’s normal” or whatever ... (interview)” (page 4) | |  |  | |  |  |
| **Patients had difficulty navigating the health system** | ““Hi, I’ve been trying to get in contact with Dr. X since I last was in the hospital, but nobody would put me through and nobody would leave a message—so now I am trying to get through here ...” (message)” (page 4)  ““Hi. I got a letter yesterday about an X-ray examination on the 7th of March at 09.30. This is the same day that I have a consultation with the physician at 08.30. I figure that I will also go to the laboratory for a blood test that morning, before the consultation. I just want to make sure that the consultation will not conflict with the X-ray. If I have to wait for the consultation I risk arriving too late for the X-ray appointment”. (message)” (page 4) | |  |  | |  |  |
